# Integrated Transcriptomics and Metabolomics Reveal Changes in Cell Homeostasis and Energy Metabolism in *Trachinotus ovatus* in Response to Acute Hypoxic Stress

**DOI:** 10.3390/ijms25021054

**Published:** 2024-01-15

**Authors:** Qing-Hua Wang, Ren-Xie Wu, Jiao-Na Ji, Jing Zhang, Su-Fang Niu, Bao-Gui Tang, Ben-Ben Miao, Zhen-Bang Liang

**Affiliations:** 1 College of Fisheries, Guangdong Ocean University, Zhanjiang 524088, China; wqh31016877430@163.com (Q.-H.W.); wurenxie@163.com (R.-X.W.); guangdongzhjjn@163.com (J.-N.J.); zjouzj@126.com (J.Z.); zjtbg@163.com (B.-G.T.); benben.miao@outlook.com (B.-B.M.); liangzhenbang0403@163.com (Z.-B.L.); 2 Southern Marine Science and Engineering Guangdong Laboratory, Zhanjiang 524025, China

**Keywords:** *Trachinotus ovatus*, acute hypoxic stress, transcriptomics, metabolomics, cell cycle arrest, pro-apoptosis, anti-apoptosis, energy metabolism

## Abstract

*Trachinotus ovatus* is an economically important mariculture fish, and hypoxia has become a critical threat to this hypoxia-sensitive species. However, the molecular adaptation mechanism of *T. ovatus* liver to hypoxia remains unclear. In this study, we investigated the effects of acute hypoxic stress (1.5 ± 0.1 mg·L^−1^ for 6 h) and re-oxygenation (5.8 ± 0.3 mg·L^−1^ for 12 h) in *T. ovatus* liver at both the transcriptomic and metabolic levels to elucidate hypoxia adaptation mechanism. Integrated transcriptomics and metabolomics analyses identified 36 genes and seven metabolites as key molecules that were highly related to signal transduction, cell growth and death, carbohydrate metabolism, amino acid metabolism, and lipid metabolism, and all played key roles in hypoxia adaptation. Of these, the hub genes *FOS* and *JUN* were pivotal hypoxia adaptation biomarkers for regulating cell growth and death. During hypoxia, up-regulation of *GADD45B* and *CDKN1A* genes induced cell cycle arrest. Enhancing intrinsic and extrinsic pathways in combination with glutathione metabolism triggered apoptosis; meanwhile, anti-apoptosis mechanism was activated after hypoxia. Expression of genes related to glycolysis, gluconeogenesis, amino acid metabolism, fat mobilization, and fatty acid biosynthesis were up-regulated after acute hypoxic stress, promoting energy supply. After re-oxygenation for 12 h, continuous apoptosis favored cellular function and tissue repair. Shifting from anaerobic metabolism (glycolysis) during hypoxia to aerobic metabolism (fatty acid β-oxidation and TCA cycle) after re-oxygenation was an important energy metabolism adaptation mechanism. Hypoxia 6 h was a critical period for metabolism alteration and cellular homeostasis, and re-oxygenation intervention should be implemented in a timely way. This study thoroughly examined the molecular response mechanism of *T. ovatus* under acute hypoxic stress, which contributes to the molecular breeding of hypoxia-tolerant cultivars.

## 1. Introduction

Dissolved oxygen (DO) is one of the most important environmental factors for the growth and survival of fish. When the DO concentration is less than 2 mg·L^−1^, aquatic animals will encounter hypoxic environmental challenges [1]. Hypoxic environments may have severe negative influences on marine ecosystems, such as massive fish kills [2]. Widespread fish kills will lead to economic losses of recreationally and commercially important fisheries, as well as decreased water quality [3]. In general, hypoxic environment is mainly caused by anthropogenic and natural factors. On one hand, high-density and large-scale aquaculture will augment the probability of hypoxic environment [3]. Excessive nutrient input into the aquatic environment from anthropogenic discharge usually causes water eutrophication and organic matter deposition, increasing microbial growth and reproduction, leading to further demand for oxygen in many coastal systems [4]. On the other hand, extreme climate fluctuations and environmental challenges, such as typhoons and high temperatures, may result in local hypoxic environments [5]. Overall, in recent years, the issue of fish hypoxic stress caused by high-density aquaculture, water eutrophication, and extreme environmental challenges has received increasing attention from the fishery scientific community.

Over evolutionary time, fish have developed hypoxia adaptation mechanisms [6,7]. A fish hypoxia adaptive strategy can typically be classified as acute stress response and long-term adaptation. Under acute hypoxic stress, fish first induce morphological and structural modifications of gills, which creates a larger area of contact with the aquatic environment to provide adequate oxygen exchange [8]. Second, an increase in red blood cells and hemoglobin combined with opening ATP-sensitive potassium channels increases the oxygen carrying capacity, which promotes oxygen transport and delivery during hypoxia [9]. Third, fish can reduce their oxygen consumption by decreasing exercise and metabolism; they can also cause floating head to occur so they can obtain oxygen [10]. In a long-term hypoxic environment, fish usually encounter more severe hypoxic challenges [11,12,13] and make a series of adaptive changes, including (a) inducing columnar gill filamentous protrusions to increase breathing [14], (b) increasing assisted respiration by cloaca, skin, and other organs [15], and (c) inhibiting anabolic pathways that consume ATP and activating catabolic pathways to generate ATP [16]. Taken together, hypoxia adaptation mechanisms play an important role in the adaptive evolution of fish. Notably, variable species-specific expression and metabolic patterns are expected in different fish under hypoxic stress [17], such as darkbarbel catfish (*Pelteobagrus vachelli*) [6,7,13], largemouth bass (*Micropterus salmoides*) [18,19], large yellow croaker (*Larimichthys crocea*) [20], longjaw mudsucker (*Gillichthys mirabilis*) [21], schizothoracine fish (*Schizothorax prenanti*) [22], spotted seabass (*Lateolabrax maculatus*) [23], yellow catfish (*Pelteobagrus fulvidraco*) [12,24], and pearl gentian grouper (*Epinephelus fuscoguttatus* ♀ × *Epinephelus lanceolatus*) [25].

The golden pompano (*Trachinotus ovatus*) is widely distributed in the tropical and subtropical waters of the Pacific, Indian Ocean, and Atlantic Ocean [26,27]. Due to its rapid growth, short breeding cycle, delicious taste, and high nutrient value, *T. ovatus* is highly favored by most consumers and aquaculture farmers [26]. As such, it is an important economic mariculture fish in China, especially in Fujian, Guangdong, Guangxi, and Hainan [27]. Over the past few years, the scale and production of *T. ovatus* aquaculture has been expanding. The aquaculture yield in China increased from 45 × 10^3^ tons in 2019 to 245 × 10^3^ tons in 2022 [28]. However, *T. ovatus* is a hypoxia-sensitive fish with higher DO requirements for survival (above 5.0 mg·L^−1^) [11,26,27], and this species is more vulnerable to hypoxic injury [26,29]. Thus, high-density aquaculture, water deterioration and eutrophication, typhoons, and high temperatures easily increase the risk of hypoxia in *T. ovatus*, thereby causing significant losses to aquaculture industry. Furthermore, hypoxia challenges not only have a direct impact on the survival of wild *T. ovatus*, they can also indirectly affect higher-level consumers and even alter trophic structure of the ecosystems to which they belong. 

Previous hypoxia studies of *T. ovatus* mainly focused on the detection of physiological and biochemical-related indices on gills and liver [11,27], as well as transcriptomic research on the gills [29]. These studies found that hypoxia had an impact on the morphological structure, physiological functions, and immune system of *T. ovatus*, eventually leading to metabolic disorders, energy imbalance, and death. Additionally, hypoxia could cause varying degrees of injury to *T. ovatus* gills and liver [11,27]. Previous studies have reported that different expression patterns and metabolic responses occur in different tissues (e.g., brain, liver, and gills) of *S. prenanti* [22] and *P. fulvidraco* [24], implying that tissue-specific expression patterns can also be expected in *T. ovatus*. The liver regulates many vital physiological processes in fish and plays a critical role in hypoxic stress responses [18,23,25]. Therefore, it is necessary to explore the molecular mechanism of *T. ovatus* liver response to acute hypoxic stress using transcriptomics and metabolomics, which is of great importance in *T. ovatus*.

In this study, we comprehensively investigated the transcriptomic and metabolic responses of *T. ovatus* liver to acute hypoxic stress and re-oxygenation using Illumina RNA sequencing (RNA-Seq) technology and liquid chromatography coupled to mass spectrometry (LC-MS) analysis. Through transcriptomics and metabolomics analyses, the key genes, metabolites, and biological pathways highly related to acute hypoxic stress were screened to elucidate the metabolic changes and clarify the molecular response mechanisms of hypoxia in the liver of *T. ovatus*. Our results would provide new insights into hypoxia adaptation in *T. ovatus*, which contributes to the molecular breeding of hypoxia-tolerant cultivars and helps cope with acute hypoxic stress.

## 2. Results

### 2.1. Transcriptomics Analysis

#### 2.1.1. Sequencing Quality and Sample Relationship

In this study, a control group (normoxia, Hy0) and three experimental groups (hypoxia 1 h, Hy1; hypoxia 6 h, Hy6; re-oxygenation 12 h, Ro12) were set up for subsequent analyses. A total of 515.18 million raw reads were obtained from 12 *T. ovatus* liver samples (Table 1), which have been uploaded to NCBI database (accession number PRJNA996362). After filtering the low-quality data, we obtained a total of 506.82 million clean reads, accounting for 98.38% of the raw reads. There were 38.28–58.25 million clean reads in the Hy0, Hy1, Hy6, and Ro12 groups, and the number of clean bases ranged from 5.72 to 8.70 Gbps. The GC content ranged from 49.00% to 49.87% and was normally distributing in the sequences of each sample, and the Q20 and Q30 values were 97.41–97.90% and 92.53–94.02%, respectively. Finally, about 506.55 million high-quality reads were generated after removing the clean reads mapped to rRNA. A total of 89.73–94.28% high-quality reads were mapped to the *T. ovatus* reference genome sequence in each sample, and 85.81–89.84% high-quality reads were uniquely mapped. Through assembly, a total of 22,814 genes were obtained, comprising 21,915 known genes and 899 novel genes. These results indicate successful construction for the cDNA library and good-quality sequencing. Moreover, no significant separation was observed among intra-group samples in the principal component analysis (PCA) (Figure S1A), and the Pearson’s correlation coefficient for pairwise comparisons between intra-group samples ranged from 0.84 to 0.97 (Figure S1B); thus, together, these results demonstrate good experimental repeatability. The above results indicate that our transcriptomics data was sufficient to continue with the following analysis.

#### 2.1.2. Identification of Differentially Expressed Genes (DEGs)

**Figure 1:**
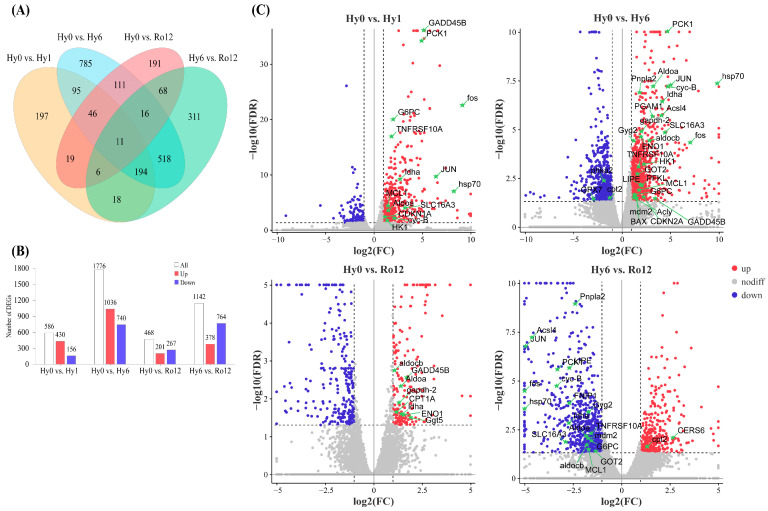
Differential expression analysis. Venn diagram (**A**) of differentially expressed genes (DEGs) in different comparisons. Bar graph (**B**) and volcano plot (**C**) showing up-regulated and down-regulated genes in different comparisons.

Differential expression analysis was implemented between four groups that identified a total of 2586 DEGs (Figure 1A; Supplementary Data S1). Specifically, the total DEGs were 586 (430 up- and 156 down-regulated) for Hy0 vs. Hy1 group, 1776 (1036 up- and 740 down-regulated) for Hy0 vs. Hy6 group, 468 (201 up- and 267 down-regulated) for Hy0 vs. Ro12 group, and 1142 (378 up- and 764 down-regulated) for Hy6 vs. Ro12 group (Figure 1B). The number of DEGs increased with time under hypoxia during acute hypoxic stress. After re-oxygenation, the number of DEGs in the Hy0 vs. Ro12 group significantly decreased compared to the Hy0 vs. Hy6 group but presented no significant difference with the Hy0 vs. Hy1 group (Figure 1C). This suggests that the mRNA level of some genes was restored after re-oxygenation, but many genes were still differentially expressed after re-oxygenation 12 h compared to that under hypoxia 0 h.

#### 2.1.3. Gene Expression Trends

**Figure 2:**
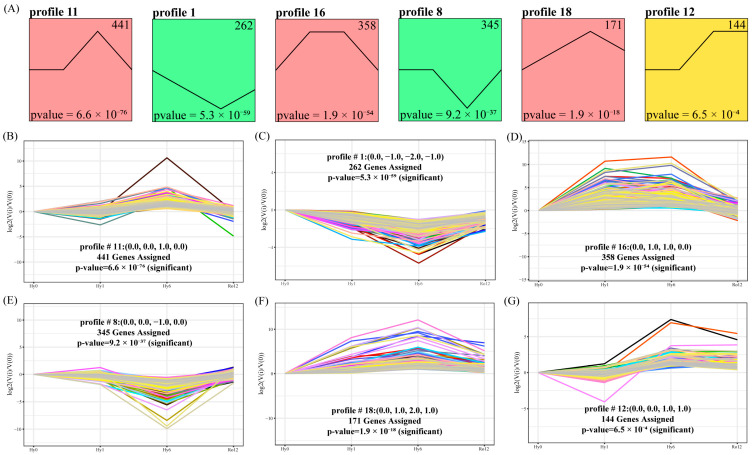
Gene expression trend analysis. (**A**) Six statistically significant gene expression profiles were detected (*p* < 0.05). Significant gene expression trends in profile 11 (441 DEGs, *p* = 6.6 × 10^−76^) (**B**), profile 1 (262 DEGs, *p* = 5.3 × 10^−59^) (**C**), profile 16 (358 DEGs, *p* = 1.9 × 10^−54^) (**D**), profile 8 (345 DEGs, *p* = 9.2 × 10^−37^) (**E**), profile 18 (171 DEGs, *p* = 1.9 × 10^−18^) (**F**), and profile 12 (144 DEGs, *p* = 6.5 × 10^−4^) (**G**).

A total of 2586 DEGs were clustered into 20 profiles, of which six gene expression profiles (profiles 11, 1, 16, 8, 18, and 12, comprising 1721 genes) were statistically significant (*p* < 0.05) (Figure 2A). Among these, 441 DEGs in profile 11 (*p* = 6.6 × 10^−76^) were significantly up-regulated at hypoxia 6 h, and then significantly down-regulated at re-oxygenation 12 h (Figure 2B). The expression levels of 262 DEGs in profile 1 (*p* = 5.3 × 10^−59^) were significantly down-regulated at hypoxia 1 and 6 h and then significantly up-regulated at re-oxygenation 12 h (Figure 2C). In profile 16 (*p* = 1.9 × 10^−54^), 358 DEGs showed significant up-regulation at hypoxia 1–6 h and significant down-regulation at re-oxygenation 12 h (Figure 2D). Comparatively, the expression levels of 345 DEGs in profile 8 (*p* = 9.2 × 10^−37^) showed an opposite trend to profile 11, which were significantly down-regulated at hypoxia 6 h and significantly up-regulated at re-oxygenation 12 h (Figure 2E). The expression levels of 171 DEGs in profile 18 (*p* = 1.9 × 10^−18^) showed an opposite trend to profile 1 that were significantly up-regulated at hypoxia 1 and 6 h and then significantly down-regulated at re-oxygenation 12 h (Figure 2F). In addition, 144 DEGs in profile 12 (*p* = 6.5 × 10^−4^) presented a significant up-regulation at hypoxia 6 h and re-oxygenation 12 h (Figure 2G). Overall, 1721 DEGs in six significant profiles were significantly up- or down-regulated at acute hypoxic stress 6 h.

#### 2.1.4. Gene Co-Expression Network Construction

**Figure 3:**
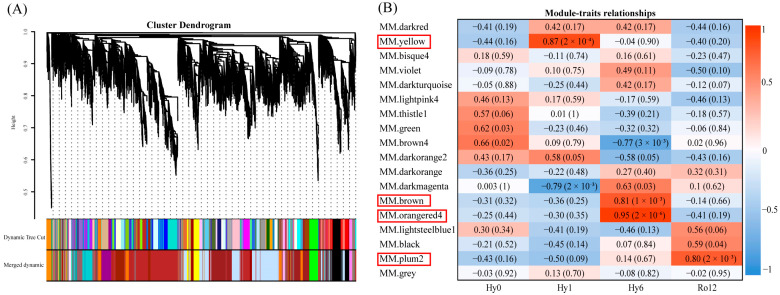
(**A**) Cluster dendrogram of 9888 genes. In total, 18 co-expression modules are shown in distinctive colors. (**B**) Module–trait relationships. The horizontal axis represents trait groups, and the longitude axis shows the 18 modules.

A clustering dendrogram of 9888 genes was constructed with dissimilarity based on topological overlap, resulting in 18 co-expression modules (Figure 3A). The number of genes in each module ranged from 5 to 2215 (Table S1). Among these modules, the yellow (*R* = 0.87, *p* = 2 × 10^−4^), brown (*R* = 0.81, *p* = 1 × 10^−3^), orangered4 (*R* = 0.95, *p* = 2 × 10^−6^), and plum2 (*R* = 0.80, *p* = 2 × 10^−3^) modules were highly correlated (Figure 3B). Specifically, 344 genes in the yellow module were highly correlated with hypoxia 1 h, 2648 genes in the brown module and orangered4 module were highly related to hypoxia 6 h, and 89 genes in the plum2 module were highly correlated with re-oxygenation 12 h. Notably, the number of highly correlated genes at hypoxia 6 h was significantly higher than that at hypoxia 1 h and re-oxygenation 12 h.

#### 2.1.5. Kyoto Encyclopedia of Genes and Genomes (KEGG) Enrichment Analysis

**Figure 4:**
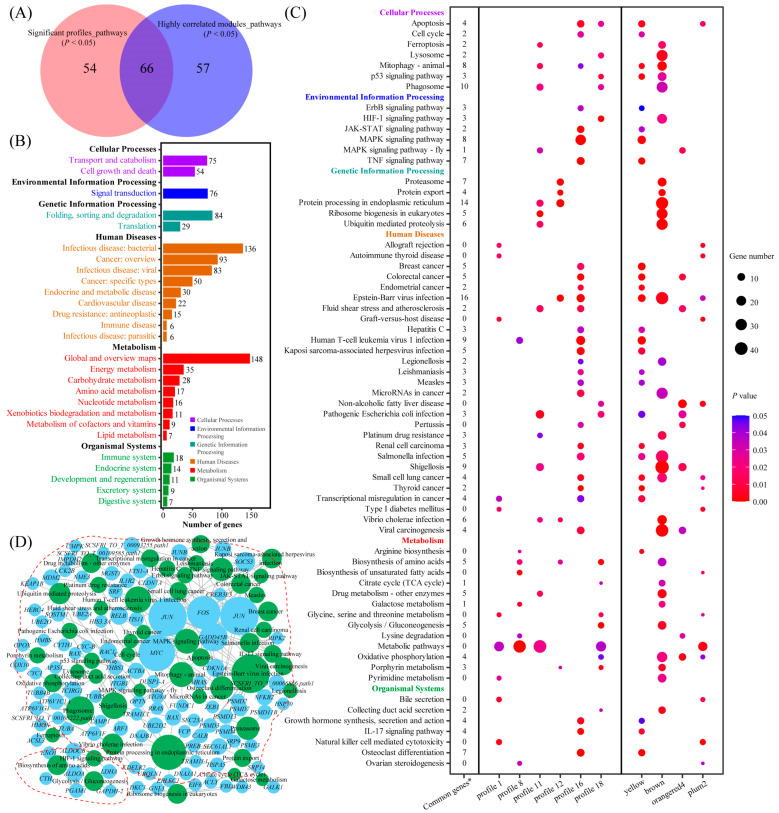
KEGG enrichment analysis. (**A**) Venn diagram of significantly enriched pathways (*p* < 0.05) between six significant gene expression profiles (red circles) and four highly correlated modules (blue circles). (**B**) KEGG classification of 66 common pathways. (**C**) The 66 common pathways of the significant profiles and highly correlated modules. * Common genes: genes simultaneously occur in both the profile-pathway and module-pathway, and the number of common genes corresponds to each pathway on the ordinate. (**D**) Pathway–gene interaction network of 66 common pathways and 103 common genes. Disconnected pathways are hidden in the network. Green circles represent pathways, and cyan circles represent genes. The size of the circle indicates the connectivity, with larger circles representing higher gene/pathway connectivity.

In the KEGG enrichment analysis, 10–52 pathways were significantly enriched in six significant gene expression profiles and four highly correlated modules. There were 66 pathways commonly enriched in both the significant gene expression profiles and highly correlated modules (Figure 4A). These pathways consisted of 625 genes (Figure 4B), of which 121 were related to cellular processes, such as apoptosis, cell cycle, and p53 signaling pathway; 76 were related to environmental information processing, such as HIF-1 signaling pathway, MAPK signaling pathway, and TNF signaling pathway; 113 were related to genetic information processing, such as proteasome, protein processing in endoplasmic reticulum, and ubiquitin mediated proteolysis; 287 were related to human diseases, such as Epstein-Barr viral infection, Human T-cell leukemia virus 1 infection, and shigellosis; 204 were related to metabolism, such as biosynthesis of amino acids, glycolysis/gluconeogenesis, and oxidative phosphorylation; and 51 were related to organismal systems, such as growth hormone synthesis, secretion and action, IL-17 signaling pathway, and osteoclast differentiation (Figure 4C). Through KEGG analysis integration, a total of 103 common genes were found in both the significant gene expression profiles and highly correlated modules (Figure 4C). Next, a pathway–gene interaction network was constructed of 66 common pathways and 103 common genes (Figure 4D). The network showed two main clusters of pathway–gene interactions: one cluster was mainly related to transport and catabolism; cell growth and death; signal transduction; and folding, sorting and degradation. The other cluster was mainly related to carbohydrate metabolism and amino acid metabolism.

#### 2.1.6. Gene Ontology (GO) Enrichment Analysis

For the GO enrichment analysis, 179–583 GO terms were significantly enriched in six significant gene expression profiles and four highly correlated modules. In total, 686 terms were commonly enriched in both the significant gene expression profiles and highly correlated modules (Figure S2A), specifically 549 terms in the biological process (BP) category, 77 terms in the cellular component (CC) category, and 60 terms in the molecular function (MF) category (Figure S2B). Of these, the most enriched GO terms for the BP category were metabolic process (GO:0008152), single-organism process (GO:0044699), and biological regulation (GO:0065007); the CC category mainly involved cell (GO:0005623), organelle (GO:0043226), and cell part (GO:0044464); and MF category mainly comprised catalytic activity (GO:0003824) and binding (GO:0005488) (Figure S2C).

#### 2.1.7. Protein–Protein Interaction (PPI) Network Analysis

**Figure 5:**
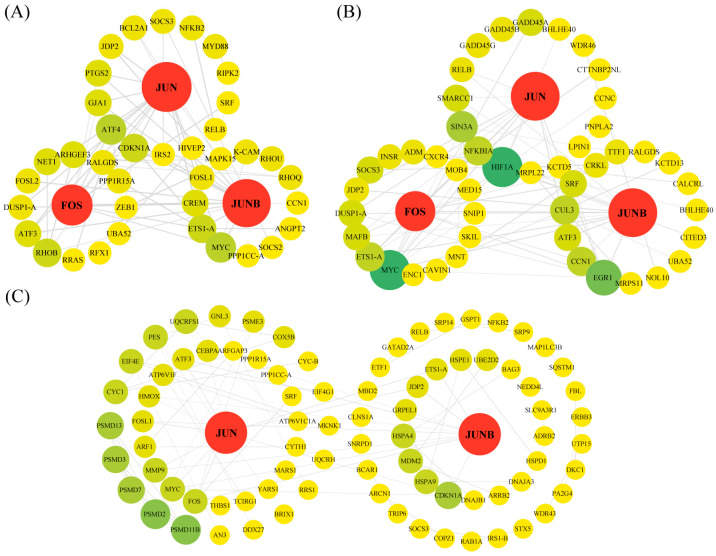
Protein–protein interaction (PPI) network analysis. The visualization of the first 100 interactions of profile 16 (**A**), yellow module (**B**), and common interactions between significant profiles and highly correlated modules (**C**). The nodes represent genes; nodes size and color are related to the degree attribute of the PPI network; and larger size and deeper color indicate higher connectivity between genes. The edges indicate the connectivity between genes; the size of the edges is correlated with the combined score attribute, with a larger size representing a higher combined score.

PPI network analysis was carried out using all genes from the six significant gene expression profiles and four highly correlated modules (Figure 5). The first 100 protein interaction pairs were used to construct the gene co-expression networks, and the genes in the PPI networks with the highest connectivity were defined as ‘hub genes’ (red circles). Specifically, the profiles and modules comprised the following: profile 1: 78 nodes, 2 hub genes (*PRKG1* and *HACE1*) (Figure S3A); profile 8: 95 nodes, 2 hub genes (*KAT2B* and *HDAC8*) (Figure S3B); profile 11: 76 nodes, 2 hub genes (*PES* and *GNL3*) (Figure S3C); profile 12: 49 nodes, 2 hub genes (*SEC61AL1* and *HSPA5*) (Figure S3D); profile 16: 41 nodes, 3 hub genes (*FOS*, *JUN*, and *JUNB*) (Figure 5A); profile 18: 63 nodes, 4 hub genes (*LDHA*, *ACLY*, *HK1*, and *ALDOA*) (Figure S3E); yellow module: 50 nodes, 3 hub genes (*FOS*, *JUN*, and *JUNB*) (Figure 5B); brown module: 89 nodes, 2 hub genes (*SEM1* and *PSMD7*) (Figure S3F); orangered4 module: 84 nodes, 2 hub genes (*RAC1* and *RHOAB*) (Figure S3G); and plum2 module: 44 nodes, 2 hub genes (*SDHB* and *APOB*) (Figure S3H). Furthermore, we constructed a gene co-expression network using the common genes between the six significant gene expression profiles and the four highly correlated modules, which contained 84 nodes and two hub genes (*FOS*, *JUNB*) (Figure 5C).

### 2.2. Metabolomics Analysis

#### 2.2.1. Metabolite Identification and Multivariate Statistical Analysis

A total of 1089 and 523 known metabolites were identified from positive and negative ion modes, respectively (Table S2). In an unsupervised statistical process, PCA analysis, including quality control (QC) samples, showed that no obvious separations were found in the intra-group samples of both positive and negative ion modes (Figure S4), suggesting a good experimental repeatability and an excellent stability and reliability of analysis system. As summarized in Figure S5 and Table S3, the parameters *R*^2^*Y* and *Q*^2^*Y* of the Orthogonal projection to latent structure-discriminant analysis (OPLS-DA) models in other comparisons in positive and negative ion modes were above 0.5, indicating the reliable predictions and the robust models; the parameter *Q*^2^*Y* of OPLS-DA model was above 0.4 in Hy0 vs. Hy1 comparison in positive ion mode, showing that the predictive abilities were generally within acceptable ranges. The quality values *Q*^2^ and *R*^2^ of permutation test were all lower than 1 in positive and negative ion modes, showing the validity and accuracy of OPLS-DA models (Figure S5).

#### 2.2.2. Identification of Differentially Expressed Metabolites (DEMs)

**Figure 6:**
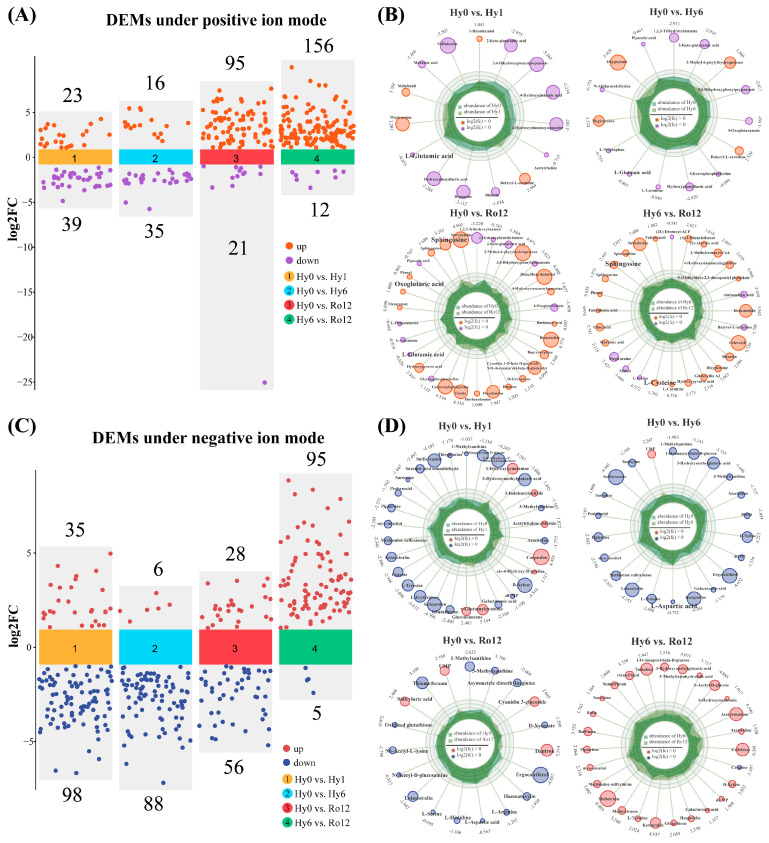
Scatter plot (**A**) and radar plot (**B**) of differential expressed metabolites (DEMs) in different comparisons in positive ion mode. Scatter plot (**C**) and radar plot (**D**) of DEMs in different comparisons in negative ion mode. Radar plot shows known metabolites.

In positive ion mode, there were 62 (23 up- and 39 down-regulated), 51 (16 up- and 35 down-regulated), 116 (95 up- and 21 down-regulated), and 168 (156 up- and 12 down-regulated) DEMs in Hy0 vs. Hy1, Hy0 vs. Hy6, Hy0 vs. Ro12, and Hy6 vs. Ro12 comparisons, respectively (Figure 6A,B; Supplementary Data S2). In negative ion mode, the total DEMs were 133 (35 up- and 98 down-regulated), 94 (6 up- and 88 down-regulated), 84 (28 up- and 56 down-regulated), and 100 (95 up- and 5 down-regulated) in Hy0 vs. Hy1, Hy0 vs. Hy6, Hy0 vs. Ro12, and Hy6 vs. Ro12 comparisons, respectively (Figure 6C,D; Supplementary Data S3). Overall, a total of 492 DEMs were identified from metabolomics analysis, including 254 and 238 DEMs from positive and negative ion modes, respectively.

#### 2.2.3. KEGG Enrichment Analysis of DEMs

**Figure 7:**
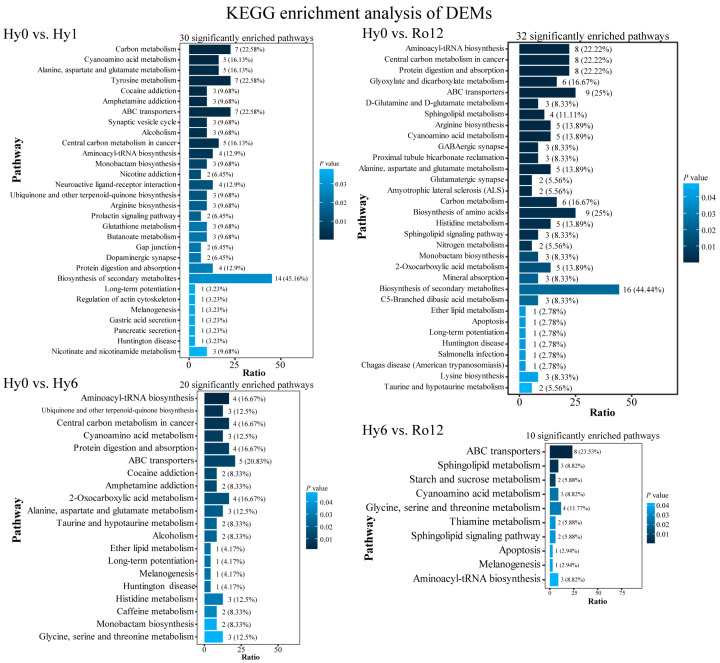
KEGG enrichment analysis of DEMs in four comparisons. Total 30, 20, 32, and 10 pathways were significantly enriched in Hy0 vs. Hy1, Hy0 vs. Hy6, Hy0 vs. Ro12, and Hy6 vs. Ro12 comparisons, respectively.

KEGG pathway enrichment analysis of DEMs in four comparison groups were performed. A total of 10–32 pathways were significantly enriched in four comparisons (Figure 7), including alanine, L-aspartic acid and glutamate metabolism, aminoacyl-tRNA biosynthesis, apoptosis, carbon metabolism, glycine, serine and threonine metabolism, histidine metabolism, and sphingolipid metabolism. These pathways were mainly related to cell growth and death, membrane transport, signal transduction, translation, amino acid metabolism, biosynthesis of other secondary metabolites, lipid metabolism, digestive system, and endocrine system.

### 2.3. Integrated KEGG Enrichment Analysis of Transcriptomics and Metabolomics

**Figure 8:**
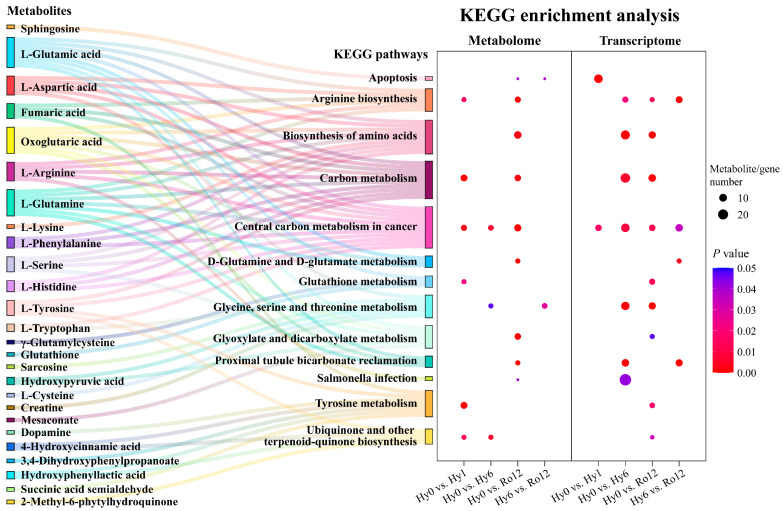
Integrated KEGG enrichment analysis of transcriptomics and metabolomics. Sankey plot (**left**) showing the relationship between DEMs and KEGG pathways, and bubble plot (**right**) showing the common significantly enriched pathways.

Significant pathways of DEGs in combination with significant pathways of DEMs were used for integrated KEGG pathway enrichment analysis. As shown in Figure 8, a total of 13 pathways were common significantly enriched, including apoptosis, arginine biosynthesis, biosynthesis of amino acids, carbon metabolism, central carbon metabolism in cancer, D-glutamine and D-glutamate metabolism, glutathione metabolism, glycine, serine and threonine metabolism, glyoxylate and dicarboxylate metabolism, proximal tubule bicarbonate reclamation, salmonella infection, tyrosine metabolism, and ubiquinone and other terpenoid-quinone biosynthesis. These pathways were mainly related to cell growth and death, amino acid metabolism, carbohydrate metabolism, and lipid metabolism. The Sankey diagram of correlation between DEMs and pathways showed that a single mapped relationship was a unique metabolite and unique pathway, e.g., sphingosine involved in apoptosis; multiple mapped relationship was in multiple metabolites and unique pathway, e.g., L-glutamic acid, γ-glutamylcysteine, and glutathione common involved in glutathione metabolism; and multiple mapped relationship was in single metabolite and multiple pathways, e.g., L-aspartic acid simultaneously involved in arginine biosynthesis, biosynthesis of amino acids, carbon metabolism, and central carbon metabolism in cancer. These results suggested that metabolites and pathways interconnected with each other and formed a complex network.

### 2.4. Key Genes, Metabolites, and Biological Pathways

Transcriptomics analysis revealed some key pathways (apoptosis, MAPK signaling pathway, ubiquitin mediated proteolysis, and glycolysis/gluconeogenesis) and biological processes (apoptotic process (GO:0006915), MAPK cascade (GO:0043408), regulation of apoptotic process (GO:0042981), and carbohydrate metabolic process (GO:0005975)), suggesting that apoptosis and metabolism play a critical role in the response to acute hypoxic stress and re-oxygenation. PPI analysis revealed some important hub genes related to signal transduction, cell growth and death, and carbohydrate metabolism. Integrated KEGG enrichment analysis of transcriptomics and metabolomics also identified some important metabolites related to cell growth and death, lipid metabolism, and amino acid metabolism. Therefore, we mainly explored transcriptomic and metabolic changes related to signal transduction, cell growth and death, carbohydrate metabolism, amino acid metabolism, and lipid metabolism in response to acute hypoxic stress and re-oxygenation.

**Figure 9:**
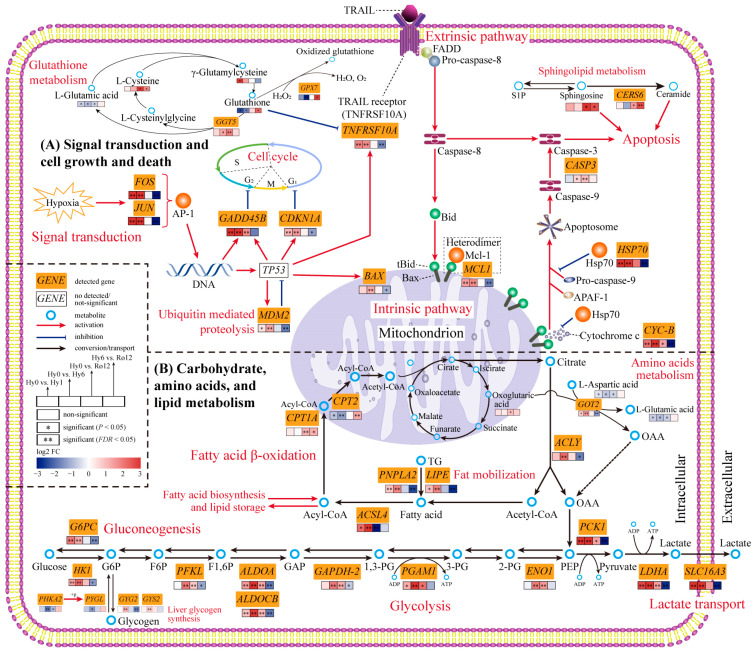
Transcriptomic and metabolic change network. Expression and change of genes and metabolites related to signal transduction and cell growth and death (**A**) as well as carbohydrate metabolism, amino acid metabolism, and lipid metabolism (**B**) in the *T. ovatus* liver in response to acute hypoxic stress and re-oxygenation.

Based on transcriptomics and metabolomics analyses, a total of 36 genes and seven metabolites were screened for further exploration in this study (Table 2). Specifically, two genes were involved in signal transduction (*FOS* and *JUN*); two genes were involved in cell cycle arrest (*GADD45B* and *CDNK1A*); seven genes and five metabolites were related to pro-apoptosis (*BAX*, *CYC-B*, *CAPS3*, *TNFRSF10A*, *GPX7*, *GGT5*, *CERS6*, glutathione, L-glutamic acid, γ-glutamylcysteine, L-cysteine, and sphingosine); three genes were related to anti-apoptosis (*MCL1*, *HSP70*, and *MDM2*); 15 genes were involved in glycolysis (*HK1*, *PFKL*, *ALDOA*, *ALDOCB*, *GAPDH-2*, *PGAM1*, *ENO1*, and *LDHA*), lactate transport (*SLC16A3*), gluconeogenesis (*G6PC* and *PCK1*), and liver glycogen synthesis (*GYG2*, *GYS2*, *PHKA2*, and *PYGL*); one gene (*GOT2*) and one metabolite (L-aspartic acid) were involved in amino acid metabolism; six genes were involved in fat mobilization (*PNPLA2* and *LIPE*), fatty acid biosynthesis (*ACSL4* and *ACLY*), and fatty acid β-oxidation (*CPT1A* and *CPT2*); and one metabolite was related to the TCA cycle (oxoglutaric acid). Finally, by further exploring the functions and pathways of these 36 genes and seven metabolites, we constructed a transcriptomic and metabolic change network to reveal the molecular mechanism in the *T. ovatus* liver in response to acute hypoxic stress and re-oxygenation (Figure 9).

### 2.5. Key Gene Expression Analysis during Acute Hypoxic Stress and Re-Oxygenation

**Figure 10:**
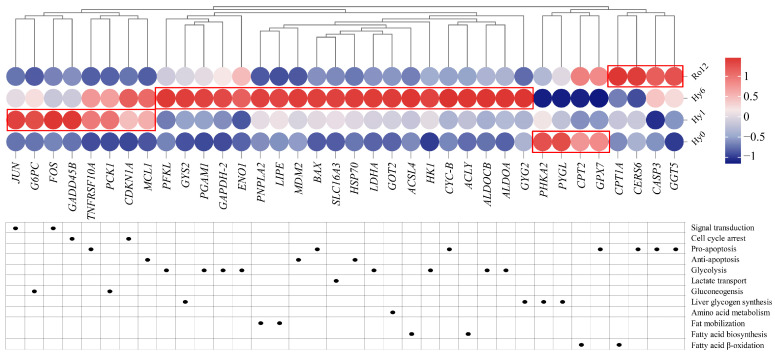
Expression heatmap of 36 key genes in *T. ovatus* under acute hypoxic stress and re-oxygenation.

Based on liver transcriptome analysis, a heatmap of the expression levels of 36 key genes identified from the present study was constructed to evaluate hypoxia damage in *T. ovatus* (Figure 10). Among these, 8 and 20 genes were significantly up-regulated after acute hypoxic stress 1 and 6 h, respectively, which indicated that the biological processes associated to these gene sets, including signal transduction, cell cycle arrest, pro-apoptosis, anti-apoptosis, glycolysis, lactate transport, gluconeogenesis, liver glycogen synthesis, amino acid metabolism, fat mobilization, and fatty acid biosynthesis, were gradually activated after acute hypoxic stress. After re-oxygenation 12 h, the above genes and biological processes gradually returned to normoxia or near normoxia levels. However, the expression level of four genes related to fatty acid β-oxidation and pro-apoptosis still remained statistically significant after re-oxygenation 12 h, suggesting that more time is needed to repair hypoxia-induced damage.

### 2.6. Quantitative Real-Time PCR (qRT-PCR) Validation of Key Genes

**Figure 11:**
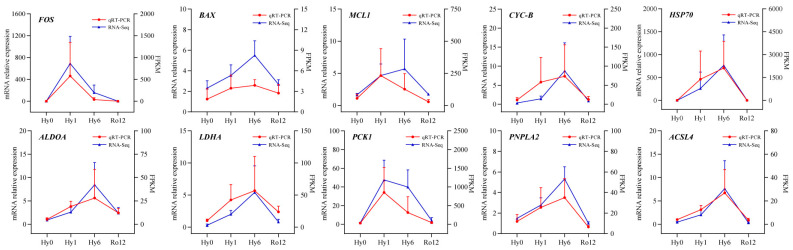
qRT-PCR validation of ten key genes from the RNA-Seq data. The mRNA relative expression values (n = 3) and FPKM values (n = 3) were calculated by comparing the hypoxia groups (Hy1 and Hy6) and re-oxygenation group (Ro12) with the control group (Hy0). qRT-PCR and FPKM data are shown as mean ± SD.

qRT-PCR was then used to validate the relative mRNA expression levels of ten key genes involved in signal transduction (*FOS*), cell growth and death (*BAX*, *MCL1*, *CYC-B*, and *HSP70*), carbohydrate metabolism (*ALDOA*, *LDHA*, and *PCK1*), and lipid metabolism (*PNPLA2* and *ACSL4*) (Figure 11). In general, the relative expression levels of ten qRT-PCR quantified genes were consistent with the RNA-Seq data, which supports the validity of our transcriptomic results.

## 3. Discussion

Through transcriptomics and metabolomics analyses, a total of 2586 DEGs and 492 DEMs were identified from *T. ovatus* liver, of which 36 genes and seven metabolites were screened as possible key molecules. These key molecules were mainly related to signal transduction, cell growth and death, carbohydrate metabolism, amino acid metabolism, and lipid metabolism, which play critical roles in hypoxia adaptation. Here, we explored the change trend in the expression of 43 key molecules and altered biological processes to deeply reveal the molecular response mechanism of *T. ovatus* under acute hypoxic stress.

### 3.1. Hub Genes in Response to Hypoxia Adaptation

In most cells, the *FOS* and *JUN* genes are regarded as important immediate-early genes in response to various stress signals [30]. In this study, expression of both genes was significantly up-regulated in the *T. ovatus* liver after acute hypoxic stress 1 and 6 h. The *FOS* and *JUN* genes encode the c-FOS and c-JUN proteins, respectively, that can dimerize to constitute the transcription factor complex activator protein-1 (AP-1) [31]. AP-1 can activate a number of hypoxia-adaptive processes related to differentiation, proliferation, and apoptosis to help cells respond to hypoxic stress [32]. For example, AP-1 plays an important role in the transcription of the growth arrest and DNA damage-inducible (Gadd45) gene family, which is beneficial for inhibiting cell cycle progression [33]. It is also an activation factor for the tumor suppressor gene *TP53*, triggering cell cycle arrest and apoptotic cell death [34]. Under hypoxic conditions, expression of the *FOS* and *JUN* genes in the gill, liver, and muscle tissues of other fish were also significantly up-regulated [6,7,35]. Therefore, the *FOS* and *JUN* genes may be two pivotal hypoxia adaptation genes, regulating cell growth and death in the liver of *T. ovatus* to adapt to a hypoxic environment.

### 3.2. Cell Growth and Death in Response to Acute Hypoxic Stress and Re-Oxygenation

#### 3.2.1. Cell Cycle Arrest during the Hypoxia Stage

Hypoxic stress has been shown to have substantial influence on the cell cycle control [33]. Our results show that cell cycle arrest-related genes (*GADD45B* and *CDKN1A*) were significantly up-regulated in the *T. ovatus* liver during acute hypoxic stress. The Gadd45 proteins, including Gadd45α, Gadd45β, and Gadd45γ, are important Cdc2/CyclinB kinase inhibitors that play a critical role in the G2 phase of cell cycle checkpoints [33]. CDKN1A, a bifunctional protein, is a control factor in the G1 phase that contributes to cell cycle progression inhibition in response to various stress signals [36]. In this study, after acute hypoxic stress, up-regulated expression of *GADD45B* and *CDKN1A* genes promoted arrest of the G1 and G2 phases of the cell cycle in the liver of *T. ovatus*. Similarly, cell cycle arrest-related genes (such as *GADD45*, *GADD45A*, and *CDKN1B*) were also reported to be significantly up-regulated in *L. maculatus* [23] and *P. vachelli* [7] during hypoxia. Arresting the cell cycle process can provide enough time to repair DNA damage [37], which can protect cells from the damaged signals and facilitate the maintenance of energy supply [38]. Therefore, cell cycle arrest and DNA repair are vital cellular protective mechanisms that contribute to maintaining genome stability and reducing energy consumption in the *T. ovatus* liver under hypoxic conditions.

#### 3.2.2. Balance of Both Pro-Apoptosis and Anti-Apoptosis Processes under Acute Hypoxic Stress

Apoptosis is a programmed and natural cell death mechanism that plays an important role in the development and homeostasis of living organisms [23,25]. In this study, four genes (*BAX*, *CYC-B*, *CASP3*, and *TNFRSF10A*) and one metabolite (glutathione) were screened out as key molecules in apoptotic process. These key molecules are directly or indirectly involved in intrinsic (mitochondria-mediated) and extrinsic (receptor-mediated) cell apoptosis pathways, playing an essential role in cellular homeostasis in the liver of *T. ovatus* after acute hypoxic stress.

In intrinsic pathway, various apoptosis-induced signals, such as the *TP53* gene activating pro-apoptosis protein Bax [39] and the death receptor activating signal molecule BH3 interacting domain death agonist (Bid) [40], can directly or indirectly alter mitochondrial membrane permeability to activate mitochondria-mediated cell apoptosis. Expression of the pro-apoptosis protein Bax causes a significant increase in mitochondrial membrane permeability and the release of cytochrome c [41]. Cytochrome c can then assemble to form the apoptosome along with apoptotic protease-activating factor (APAF-1) and pro-caspase-9 in the cytoplasm [42]. Subsequently, pro-caspase-9 is cleaved in the apoptosome, producing activated caspase-9, which finally cleaves and activates down-stream caspases (such as caspase-3) to trigger apoptosis [40]. In this study, during acute hypoxic stress, up-regulation of *BAX*, *CYC-B*, and *CASP3* genes in the liver of *T. ovatus* can activate intrinsic pathway and promote apoptosis progression. Under hypoxic conditions, similar up-regulation of pro-apoptosis related genes (such as *BAX*, *CASP9*, and *CASP3*) has also been reported in *P. vachelli* [7,13] and *L. maculatus* [23].

In extrinsic pathways, the death receptors are usually activated when they bind to their corresponding cognate ligand. For example, the tumor necrosis factor (TNF)-related apoptosis-inducing ligand (TRAIL) can bind to its receptor (TNFRSF10A), triggering trimerization of the receptors [43]. This receptor trimerization recruits and binds the Fas-associated death domain (FADD) and pro-caspase-8, thereby forming the death-inducing signaling complex (DISC) [40]. The formation of DISC activates caspase-8, which directly cleaves the down-stream caspase-3 (encoded by *CASP3* gene) or indirectly activates the intrinsic pathway by inducing the expression of Bid, which triggers apoptotic cell death [40]. In this study, up-regulation of the *TNFRSF10A* and *CASP3* genes in the liver of *T. ovatus* under hypoxic conditions can trigger the recruitment of TRAIL to bind to its receptor and activate the extrinsic pathway. In previous studies, death receptor related genes (*TNFRSF10* and *TNFRSF19*) were also up-regulated in the gills of *T. ovatus* during hypoxia [29], which suggests that hypoxic damage induces cell apoptosis in different tissues [11,27].

Glutathione is an important non-protein sulfhydryl compound that is synthesized from L-glutamic acid, L-cysteine, and glycine, playing a critical role in apoptosis [44]. The glutathione related antioxidant gene *GPX7* encodes an enzyme glutathione peroxidase 7 that catalyzes glutathione and H_2_O_2_ to oxidized glutathione, H_2_O, and O_2_ in the glutathione redox system, thus preventing an adverse influence of oxidative stress on cells [45]. The *GGT5* is an important genes for encoding glutathione hydrolase, which promotes the hydrolysis of glutathione into L-glutamic acid, L-cysteine, and glycine [46]. In our study, down-regulation of the *GPX7* gene and up-regulation of the *GGT5* gene suggested that the glutathione oxidation process was inhibited while the glutathione hydrolase process was enhanced in the liver of *T. ovatus* during acute hypoxic stress. In line with transcriptome analysis, the content of glutathione and L-glutamic acid were decreased while γ-glutamylcysteine content was increased under hypoxia condition. Similar findings, that glutathione metabolism was enhanced by hypoxic stress, have also been found in *S. prenanti* [22], *P. vachelli* [13], and *E. fuscoguttatus* ♀ × *E. lanceolatus* ♂ [25]. Previous studies have reported that the decrease of glutathione content can enhance the sensitivity of death receptor (TRAIL) and induce apoptosis occurrence [44]. Therefore, decreased glutathione content could further activate the death receptor-mediated extrinsic pathway to trigger apoptosis in the liver of *T. ovatus*. Taken together, inhibiting glutathione oxidation, promoting glutathione hydrolysis, and activating extrinsic pathway all contributed to triggering the apoptotic process and maintaining cell homeostasis for hypoxia adaptation, showing the importance of glutathione metabolism in response to hypoxic stress in the liver of *T. ovatus*.

Notably, during acute hypoxic stress, when pro-apoptosis was induced, an anti-apoptosis process was also activated in the *T. ovatus* liver. This was nicely illustrated by up-regulated expression of *MDM2*, *MCL1*, and *HSP70*. *MDM2* is a critical negative regulator of the *TP53* tumor suppressor [47]. *MCL1* is an anti-apoptotic member of the Bcl-2 family proteins and can bind to cell death mediators (e.g., tBid), forming a heterodimer that inhibits apoptosis [48]. Hsp70 is usually characterized as a molecular chaperone that regulates cell apoptosis in response to oxidative stress by preventing the release of cytochrome c [49]. Hsp70 can also prevent the recruitment of pro-caspase-9 to the apoptosome complex, thus blocking the assembly of a functional apoptosome and the consequent activation of caspase-9 [50]. Therefore, under acute hypoxic stress, up-regulation of *MDM2*, *MCL1*, and *HSP70* genes was conducive to effectively inhibiting apoptosis in the *T. ovatus* liver. Similarly, during hypoxic stress, up-regulation of anti-apoptosis-related genes (such as *MCL1A*, *MCL1B*, and *BCL2*) have also been found in *P. fulvidraco* [12], *P. vachelli* [7,13], and *E. fuscoguttatus* ♀ × *E. lanceolatus* ♂ [25]. In which both pro-apoptosis and anti-apoptosis-related genes were simultaneously up-regulated, during acute hypoxic stress, when apoptotic death is activated in hypoxia-induced cells, the anti-apoptosis protective mechanism is also initiated, which protects cells to maintain stable cellular function in hypoxic environments. Taken together, the balance of pro-apoptosis and anti-apoptosis processes is a critical factor for the *T. ovatus* liver in hypoxia adaptation.

#### 3.2.3. Continuous Apoptosis after Re-Oxygenation

If fish are exposed to hypoxia stress for a long time, serious damage to cellular function usually occurs. Re-oxygenation after hypoxic stress is a traditional and effective strategy to reduce hypoxia-induced injury to fish [23]. After re-oxygenation 12 h, the gene expression of and metabolite content related to cell cycle arrest, pro-apoptosis, and anti-apoptosis gradually returned to normoxia levels after re-oxygenation 12 h. However, apoptosis was continuously induced in the liver of *T. ovatus* due to significant up-regulation of pro-apoptosis related genes (*CYC-B*, *CASP3*, and *CERS6*) and significant increasing of pro-apoptosis related metabolite (sphingosine) after re-oxygenation. Sphingomyelin metabolites, including ceramide, sphingosine, and sphingosine 1-phosphate (S1P), are a class of prominent metabolites, acting as an important regulator of cell growth and apoptosis [51,52]. Ceramide and sphingosine can induce cell growth arrest, migration, and apoptosis, while S1P promotes cell survival, differentiation, and proliferation [52]. The *CERS6* gene encodes an enzyme ceramide synthase 6 that catalyzes the conversion of sphingosine to ceramide, which plays a critical role in sphingolipid metabolism [51]. In this study, *CERS6* gene was up-regulated and sphingosine content was significantly increased after re-oxygenation 12 h, which promoted the production of ceramide. These changes combined with significant up-regulation expression of *CASP3* triggered a continuous apoptosis in *T. ovatus* liver after re-oxygenation 12 h. Continuous apoptosis after re-oxygenation is considered a critical hypoxia adaptation mechanism, in which damaged cells are cleared in response to hypoxia-induced injury during hypoxic stress that triggers the apoptotic cell death, cellular function repair, and tissue repair during re-oxygenation [40,53]. A similar mechanism of continuous apoptosis after re-oxygenation has also been found in *L. maculatus* [23] and *P. vachelli* [13].

### 3.3. Carbohydrate Metabolism, Amino Acid Metabolism, and Lipid Metabolism in Response to Acute Hypoxic Stress and Re-Oxygenation

#### 3.3.1. Enhancing Anaerobic Glycolysis and Lactate Transport for Hypoxia Adaptation

For aquatic animals, energy metabolism, including aerobic metabolism and anaerobic metabolism, always plays a critical role in response to extreme environmental changes [12,18,25]. Under hypoxic conditions, aerobic metabolism is inhibited, forcing the switch to anaerobic metabolism to produce ATP for energy requirements [54]. The glycolysis pathway is one of the most important anaerobic metabolism pathways and is well known as an oxygen-independent process that generates ATP [16]. Our transcriptomic results show that the *HK1*, *PFKL*, *ALDOA*, *ALDOCB*, *GAPDH-2*, *PGAM1*, and *ENO1* genes involved in the glycolysis pathway were significantly up-regulated after acute hypoxic stress. Up-regulation of glycolysis-related genes can promote the activation of glycolytic process and the generation of pyruvate and ATP from glucose. The main product of the glycolysis pathway, pyruvate, can be converted to acetyl-CoA, which enters the TCA cycle when oxygen is sufficient; however, under hypoxic conditions, this process is inhibited and pyruvate will be converted to the lactate by lactate dehydrogenase A (LDH-A) [55]. LDH-A (encoded by *LDHA* gene), a marker enzyme of anaerobic metabolism and has been demonstrated to catalyze the conversion of pyruvate to lactate, with its activity reflecting the intensity of anaerobic metabolism to some extent [12,27]. In this study, expression of the *LDHA* gene was significantly up-regulated at hypoxia 1 and 6 h, which is consistent with the increased LDH activity in the liver of *T. ovatus* during acute hypoxic stress [27]. Enhancement of the glycolytic effect due to the up-regulation of related genes, such as *HK1*, *PFKL*, and *LDHA* genes, in response to acute hypoxic stress have has also been found in the liver of *P. vachelli* [6], *L. crocea* [20], and *M. salmoides* [18]. In line with these previous findings, our results further support the important role of the glycolysis pathway in fish for hypoxia adaptation.

Of note, with increasing time under hypoxia, the glycolysis product lactate will accumulate in large quantities, further inhibiting the glycolytic process, decreasing pH, and even endangering life [20]. Thus, lactate transport and metabolism in a timely manner is essential for the stability of the intracellular environment. Under a hypoxic environment, lactate cannot be oxidatively metabolized in the TCA cycle [56], but it can be transported from the intracellular to extracellular spaces by the monocarboxylic acid transporter family [57]. SLC16A3, a member of the monocarboxylic acid transporter family, is responsible for transmembrane transport of multiple monocarboxylic acids, in particular the outflow of lactate [57]. Therefore, significant up-regulation of *SLC16A3* gene is beneficial for transporting excess lactate out of the cell, maintaining the homeostasis of intracellular environment. Yang et al. [19] and Ding et al. [20] also detected up-regulation of the *SLC16A3* gene in the liver of *M. salmoides* and *L. crocea*, respectively, in response to acute hypoxic stress. Therefore, timely lactate efflux and metabolism is an important biological process that enhances glycolysis to constantly provide energy for *T. ovatus* and maintain intracellular homeostasis. Taken together, the anaerobic glycolytic process and lactate transport were activated during the early stage of hypoxic stress, then continuously enhanced for the duration of long-term hypoxia, which could ensure an adaptive strategy that provides a large amount of ATP in response to long-term hypoxic stress, thereby maintaining the intracellular energy balance and homeostasis in the *T. ovatus* liver.

#### 3.3.2. Enhancing Gluconeogenesis and Glycogen Synthesis for Hypoxia Adaptation

It is worth noting that the amount of glucose stored in the liver is limited, but the continuous glycolytic process continuously consumes glucose. A lower glucose concentration may stimulate the gluconeogenesis pathway to produce endogenous glucose to meet the substrate requirements. Glucose-6-phosphatase (G-6-Pase) and phosphoenolpyruvate carboxykinase (PEPCK) are the key rate-limiting enzymes of the gluconeogenesis pathway and are encoded by the *G6PC* and *PCK1* genes, respectively [58,59]. The mRNA abundance of the *G6PC* and *PCK1* genes have been considered indicators to determine the intensity of the gluconeogenesis process [58,59]. In this study, expression of *G6PC* and *PCK1* genes was significantly up-regulated after acute hypoxic stress, suggesting that the gluconeogenesis process was enhanced in the liver of *T. ovatus*. Similarly, up-regulation of gluconeogenesis-related genes (*G6Pase* and *PEPCK*) and an increase activity in gluconeogenesis-related enzymes (G-6-Pase and PEPCK) were detected in *L. crocea* under hypoxic conditions [20]. In contrast, down-regulation of the gluconeogenesis-related gene (*FBP*) and decrease in gluconeogenesis-related enzyme activity (PEPCK) was found in *M. salmoides* after hypoxic stress [18]. These findings indicated expression-specific hypoxia adaptation in different fish. Furthermore, previous studies have reported some distinct cell populations in the teleost liver showing metabolic heterogeneity linked to the abundance of cytoplasmic mitochondrion [60]. Cell populations showing glycolytic heterogeneity with a lower mitochondrial abundance can increase the generation of energy, while those showing gluconeogenesis heterogeneity with a higher mitochondrial abundance promote the synthesis of endogenous glucose and the production of liver glycogen [60]. Based on our transcriptomic data and previous studies, we found that the hypoxic response included those heterogeneous cell populations that simultaneously undertook glycolysis and gluconeogenesis under hypoxic conditions. The key genes involved in glycolysis (e.g., *HK1*, *PFKL*, and *LDHA* genes) were up-regulated in those cell populations with a lower mitochondrial abundance to produce ATP to meet energy requirements, while those involved in gluconeogenesis (e.g., *G6PC* and *PCK1* genes) were up-regulated in cell populations with a higher mitochondrial abundance to synthesize endogenous glucose and supply the glycolysis-consumed glucose. Similar findings, that glycolysis and gluconeogenesis pathways were concomitantly promoted in distinct cell populations under hypoxic conditions, have also been reported in *P. fulvidraco* [12]. Glucose produced by the gluconeogenesis process can not only provide indispensable substrate for the initiation of glycolysis, but it may also be transformed into liver glycogen that, to some extent, can maintain the level of liver glycogen during acute hypoxic stress. Our transcriptome data shows that, under acute hypoxic stress, liver glycogen synthesis related genes (*GYG2* and *GYS2*) were up-regulated, while glycogenolysis-related genes (*PHKA2* and *PYGL*) were down-regulated, suggesting that hypoxia promoted liver glycogen synthesis and inhibited glycogenolysis to maintain the liver glycogen level [61,62,63]. Similar liver transcriptomic results have also been reported for *P. vachelli* under acute hypoxic stress [6]. Thus, the increase in the gluconeogenesis process and liver glycogen synthesis are beneficial for hypoxia adaptation in fish.

#### 3.3.3. Enhancing Amino Acid Metabolism for Hypoxia Adaption

Under acute hypoxic stress, fish usually increase their anaerobic metabolism (e.g., glycolysis) to produce ATP for energy supply and normal physiological functions [16]; when energy supply is insufficient, protein or amino acid will eventually be converted into carbohydrates to produce ATP and meet energy requirements [64]. Our study revealed that gene *GOT2* and metabolite L-aspartic acid were significantly up-regulated and decreased, respectively, after acute hypoxic stress. GOT2 acts as a key enzyme that catalyzes the conversion of L-aspartic acid and oxoglutaric acid to L-glutamic acid and Oxaloacetic acid (OAA) [65]. Decreasing L-aspartic acid content and up-regulation of *GOT2* gene indicated that amino acid metabolism was enhanced in the liver of *T. ovatus* after acute hypoxic stress. The L-aspartic acid catalyzed by the enzyme GOT2 serves as an alternative metabolite of pyruvate to generate and supply the OAA, which provides a large amount of metabolic substrates for glycolysis. The interaction between amino acid metabolism and the glycolysis process is conducive to ensure stable blood glucose balance and meet energy requirement in the organism under acute hypoxic stress, which may be an important molecular response mechanism in the liver of *T. ovatus* for hypoxia adaptation. Under hypoxic stress, similar molecular response mechanisms were also found in *G. mirabilis* [21] and *L. crocea* [20]. These shared findings indicate that amino acid metabolism helped compensating for the lack of energy supply by generating glucose and intermediate metabolites in glucose metabolism during acute hypoxic stress and was beneficial for fish that were adapting to a hypoxic environment.

#### 3.3.4. Enhancing Fat Mobilization and Fatty Acid Biosynthesis for Hypoxia Adaption

Along with carbohydrate metabolism and amino acid metabolism, lipid metabolism also plays an important role in the response to long-term acute hypoxic stress for fish. Many studies have shown that fat mobilization and fatty acid biosynthesis are increased in the liver of fish after hypoxic stress [18,25]. This was also nicely illustrated by the up-regulated expression of fat mobilization (*PNPLA2* and *LIPE*) and fatty acid biosynthesis (*ACSL4* and *ACLY*)-related genes in the liver of *T. ovatus* after acute hypoxic stress. Adipose triglyceride lipase (ATGL) and hormone-sensitive lipase (HSL), encoded by *PNPLA2* and *LIPE* genes, respectively, are the key lipolytic enzymes in fat mobilization, accounting for a majority of the triglyceride (TG) hydrolysis processes [66,67]. Therefore, up-regulation of *PNPLA2* and *LIPE* genes can promote TG hydrolysis to generate glycerol and fatty acids. Fatty acids will be converted to acyl-CoA under the role of acyl-CoA synthetase long-chain family member 4 (ACSL4), and up-regulation of *ACSL4* gene after acute hypoxic stress promotes the accumulation of activated acyl-CoA, which contributes to fatty acid biosynthesis and lipid storage [68]. The accumulation of activated acyl-CoA is an important process in preparation for possible long-term hypoxia [18]. Furthermore, ACLY is a key enzyme involved in fatty acid biosynthesis and is regarded as a bridge between glucose metabolism and fatty acid metabolism, catalyzing the production of acetyl-CoA and OAA from citrate [69]. Therefore, hypoxic stress can increase the expression of genes related to fat mobilization and fatty acid biosynthesis under acute hypoxic stress, which contributed to lipid storage and energy supply in the liver of *T. ovatus* during long-term hypoxia.

#### 3.3.5. Activating Fatty Acid β-Oxidation and Aerobic Metabolism after Re-Oxygenation

Our transcriptomic data shows that the expression of carbohydrate and lipid metabolism-related genes in the liver of *T. ovatus* were significantly changed after re-oxygenation compared with the hypoxic stress period. In terms of carbohydrate metabolism, after re-oxygenation 12 h, the expressions of genes related to glycolysis, lactate transport, gluconeogenesis, amino acid metabolism, and liver glycogen synthesis were significantly or non-significantly up-regulated compared with hypoxia 0 h, but significantly or non-significantly down-regulated compared to hypoxia 6 h, suggesting that these biological processes gradually recover in the liver of *T. ovatus*. Notably, anaerobic metabolism-related genes (such as *PFKL* and *LDHA*) were down-regulated compared to hypoxia 6 h but still significantly up-regulated compared with hypoxia 0 h, implying that a longer time was needed to restore normal physiological activity of the *T. ovatus* liver under normoxic conditions. With respect to lipid metabolism, along with the down-regulation of fat mobilization and fatty acid biosynthesis-related genes, fatty acid β-oxidation related genes (*CPT1A* and *CPT2*) were up-regulated after re-oxygenation 12 h in comparison to hypoxia 0 or 6 h, indicating a critical transformation of the energy metabolism strategy in the *T. ovatus* liver. Carnitine palmitoyl transferase 1 (CPT1A) and carnitine palmitoyl transferase (CPT2), which are key enzymes in the fatty acids β-oxidation process, allow the movement of acyl-carnitine from the cytosol into the intermembrane space of the mitochondria [70,71]. Under aerobic conditions, the end product acyl-CoA from fatty acid β-oxidation is converted to acetyl-CoA in the mitochondrion to enter the TCA cycle for aerobic metabolism [72]. Correspondingly, a key metabolite of TCA cycle process, oxoglutaric acid, was significantly increased after re-oxygenation compared to hypoxia 0 h, suggesting that TCA cycle was enhanced along with the recovery of DO. Therefore, up-regulation of *CPT1A* and *CPT2* genes can promote fatty acid β-oxidation and contribute to the activation of aerobic metabolism by the TCA cycle in the liver of *T. ovatus*. This indicates that the energy metabolism strategy mainly shifted from anaerobic metabolism (glycolysis) during hypoxia to aerobic metabolism (fatty acid β-oxidation and TCA cycle) after re-oxygenation, which may be an important adaptation mechanism in the *T. ovatus* liver.

### 3.4. Key Periods for Acute Hypoxic Stress and Re-Oxygenation in T. ovatus

Over the course of hypoxic stress experiment, we found that *T. ovatus* swam swiftly and occasionally presented floating heads when encountering hypoxic stress; after acute hypoxic stress 6 h, *T. ovatus* began to suffocate and die. These phenomena demonstrated that hypoxia 6 h was a key period for hypoxia-induced damage in *T. ovatus*, which was further suggested by the results of our transcriptome and previous studies. This study showed that signal transduction, cell growth and death, carbohydrate metabolism, amino acid metabolism, and lipid metabolism processes were significantly altered after acute hypoxic stress 6 h. Although these biological processes could largely restore after re-oxygenation 12 h, continuous apoptosis demonstrated that hypoxia 6 h has caused some degree of hypoxia-induced damage in the liver of *T. ovatus*, which has also been reported in the previous studies [11,27]. After acute hypoxic stress 6 h, histological analysis observed obvious fat vacuoles and slight degeneration of fat vesicles in *T. ovatus* liver [11]; the number of mitochondria in hepatocytes of *T. ovatus* decreased, especially the small mitochondria that disappeared directly [11]; intracellular ROS level was increased, and antioxidant system was enhanced to attenuate oxidative damage in the liver of *T. ovatus* [11]. One should note that the gill transcriptome results of *T. ovatus* were paralleled with our results [29]. Integrated mRNA-Seq and miRNA-Seq analysis of *E. fuscoguttatus* ♀ × *E. lanceolatus* ♂ also revealed that metabolic patterns and ROS level in liver were significantly altered in response to acute hypoxic stress 3–6 h, and acute hypoxic stress for 9 h caused severe oxidative damage and a significant increase in the mortality rate [73]. Considering the above analysis in combination with our results from the key gene expression analysis, we conclude that hypoxia 6 h was the critical period during which metabolism and apoptotic processes were significantly altered in *T. ovatus*. Based on the above findings, the current study concluded that acute hypoxic stress 6 h was a critical period for *T. ovatus* juveniles in terms of cellular metabolism alteration and cell homeostasis maintenance. Given this, if *T. ovatus* juveniles experience acute hypoxic stress in the process of aquaculture, re-oxygenation intervention should be implemented within 6 h so as to avoid irreversible damage to the body, further avoiding mass mortality and serious economic losses.

Re-oxygenation after hypoxia is thought to be an effective and traditional approach to alleviating hypoxia-induced damage to fish [23]. In this study, mRNA expression level of some genes (e.g., *CPT1A*, *CERS6*, *CASP3*, and *GGT5*) related to fatty acid β-oxidation and pro-apoptosis processes still remained statistically significant after re-oxygenation 12 h, suggesting that more time is needed for *T. ovatus* to fully repair oxidative damage. Gill transcriptome analysis of *T. ovatus* also showed the number of DEGs was decreased but there were still some DEGs after re-oxygenation 12 h and 24 h [29]. In liver transcriptome analysis of *M. salmoides*, re-oxygenation 12 h restored the ATP level to 62.5% of control value [18]. Together, these results strongly implicate that *T. ovatus* juveniles, having experienced acute hypoxic stress 6 h, cannot totally return to normal after re-oxygenation 12–24 h. Thus, if hypoxia has occurred, the re-oxygenation process should be performed for longer than 24 h in hypoxia-sensitive *T. ovatus* to allow them to fully recover to normal. This study reveals the critical periods for hypoxic stress and re-oxygenation of *T. ovatus* juveniles, which provide an important scientific timeframe for coping with acute hypoxic stress during the culture period.

## 4. Materials and Methods

### 4.1. Ethics Statement and Fish Management

**Figure 12:**
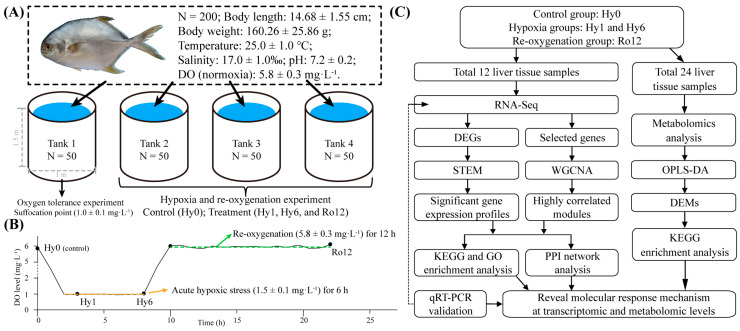
Fish management (**A**), hypoxia and re-oxygenation experimental design (**B**), and technical roadmap of transcriptomics and metabolomics analysis (**C**).

The experimental animal protocols undertaken in the present study were reviewed and approved by the Animal Experimental Ethics Committee of Guangdong Ocean University, China. A total of 200 *T. ovatus* juveniles (body length = 14.68 ± 1.55 cm, body weight = 160.26 ± 25.86 g) were purchased from Zhanjiang Hist Aquatic Technology Co., Ltd. Zhanjiang, Guangdong, China. The fish were divided over four plastic tanks (diameter = 1.0 m, height = 1.5 m; one tank for oxygen tolerance experiment and three tanks for hypoxia and re-oxygenation experiment; 50 fish per tank). All fish were acclimated to the culture environment for two weeks to minimize the stress associated with the experiment (Figure 12A). During the acclimation period, all fish were fed with commercial pellets (feed amount: 3–5% of fish body weight) twice a day (at 9:00 a.m. and 17:00 p.m.). After 1 h of feeding, food residue was artificially removed and the water (30–40%) was exchanged every day to maintain a stable water quality environment (water temperature 25.0 ± 1.0 °C, salinity 17.0 ± 1.0‰, and pH 7.2 ± 0.2). In addition, the DO level was maintained at 5.8 ± 0.3 mg·L^−1^ using an aerator (Saier, SC-60, Guangzhou, China), defined as the level of normoxia.

### 4.2. Experimental Design and Sample Collection

Our oxygen tolerance experiment determined that the suffocation point of *T. ovatus* was 1.0 ± 0.1 mg·L^−1^. Referring to our oxygen tolerance experiment results, in combination with previous studies by Chen et al. [11] and Ou et al. [27], we defined 1.5 ± 0.1 mg·L^−1^ DO as the acute hypoxic stress threshold for the formal experiment (Figure 12B). At this DO concentration, all fish were under a state of acute hypoxic stress (fish swam swiftly and occasionally presented floating heads). Fish were fasted for 24 h prior to the start of the experiment. At the onset of acute hypoxic stress, the DO level was reduced from 5.8 ± 0.3 mg·L^−1^ to 1.5 ± 0.1 mg·L^−1^ within 2 h by pumping nitrogen into the three experimental tanks using nitrogen gas cylinders (Zhanjiang Oxygen Company, Zhanjiang, China). Subsequently, the experimental fish were exposed to acute hypoxic conditions (1.5 ± 0.1 mg·L^−1^) for 6 h. After acute hypoxic stress 6 h, the nitrogen purge was stopped and oxygen was pumped into the tanks with normoxia (5.8 ± 0.3 mg·L^−1^) being reached within 2 h, followed by re-oxygenation for 12 h. For the duration of the formal experiment, all water quality parameters except for the DO were consistent with the acclimation period.

At normoxia (Hy0 group, control), hypoxia 1 and 6 h (Hy1 and Hy6 groups, respectively), and re-oxygenation 12 h (Ro12 group), six fish were randomly sampled from each tank (total 18 samples per time point) and rapidly anesthetized with MS-222 (South Ranch, Zhengzhou, China). After measuring the body length and weight, the liver tissues were rapidly and carefully dissected, transferred immediately into 1.5 mL RNase-free tubes, quickly frozen in liquid nitrogen, and finally stored in a −80 °C ultra-low temperature freezer (Haier, Qingdao, China) for further experiments.

### 4.3. Transcriptomics Analysis

We randomly selected three samples from each group (total 12 samples from four groups) to construct cDNA libraries (Figure 12C). Total RNA was extracted from liver tissues (50–100 mg) using the Trizol Reagent Kit (Invitrogen, Carlsbad, CA, USA) according to the manufacturer’s instructions, and mRNA was enriched using magnetic beads with Oligo (dT) (Qiagen, Germantown, MD, USA). The integrity, purity, and concentration of mRNA were assessed by 1% agarose gel electrophoresis and a NanoDrop 2000 Spectrophotometer (Thermo Fisher Scientific, Waltham, MA, USA). The qualified mRNA (1.8 < OD260/280 < 2.0, RIN > 8.0) was used for downstream library preparations and qRT-PCR.

The mRNA was fragmented into short fragments using a fragmentation buffer. The RNA fragments were subsequently reverse-transcribed into the first-strand cDNA using random primers, and then second-strand cDNA was synthesized using DNA polymerase I, dNTPs, RNase H, and buffer. The cDNA fragments were purified with the QiaQuick PCR extraction kit (Qiagen, Venlo, The Netherlands), end repaired, A-bases added, and ligated to Illumina sequencing adapters. Finally, approximately 200 bp cDNA were screened with AMPure XP beads to construct the cDNA library. All cDNA libraries were sequenced on the NovaSeq 6000 System (Illumina, San Diego, CA, USA) at Gene Denovo Biotechnology Co., Ltd. (Guangzhou, China).

The raw sequencing reads were visualized and checked using Fastqc v0.18.0 [74]. Clear reads were obtained by filtering the low-quality reads containing adapter-ligated contaminants and removing reads with more than 50% of low quality (Q-value < 20) bases, over 10% of poly(N), and all A-bases. The quality of the clean reads was determined by the GC content, and the Q20 and Q30 values. The clean reads were first mapped to the ribosomal RNA (rRNA) database using Bowtie v2.2.8 [75], and then the rRNA-mapped reads were removed to acquire high-quality reads. The high-quality clean reads were mapped to the *T. ovatus* reference genome [76] using Hisat v2.2.4 [77]. The mapped reads of each sample were assembled by StringTie v1.3.1 [78] using a reference-based approach, and any gene that was not included in the *T. ovatus* reference genome was defined as a ‘novel gene’. For each transcription region, the fragment per kilobase of transcript per million mapped reads (FPKM) value was calculated using RSEM [79] to quantify its expression abundance and variation. PCA and Pearson’s correlation coefficient analysis were carried out to visualize the relationships between samples and to evaluate the biological repetition correlation using R v4.0.2.

Differential expression analysis between two different groups was performed using DESeq2 [80]. Genes with the parameters of false discovery rate (*FDR*) < 0.05 and |log2 (fold change)| > 1 were considered DEGs.

The FPKM values of all DEGs were log2-transformed into preprocessed data. Subsequently, to obtain the gene expression profiles associated with acute hypoxic stress and re-oxygenation, a gene expression trend analysis was conducted with a short time-series expression miner (STEM) program [81]. The parameters for trend analysis were as follows: the maximum unit of change in the model profile between time nodes = 2 and the maximum number of model profiles = 20; the other parameters were set as default. Profiles with a threshold of *p* < 0.05 were defined as significant gene expression profiles.

A weighted gene co-expression network analysis (WGCNA) was performed to identify potential hypoxia-related gene co-expression modules. After filtering low expression genes (FPKM < 1, more than half of the samples), the FPKM values of the selected genes were imported into WGCNA v1.47 [82] to construct co-expression modules using the automatic network construction function block wise modules. The parameters were as follows: power = 9, type of topological overlap matrix (TOM) = unsigned, merge cut height = 0.25 and minimum module size = 50; the other settings were default. Gene modules with significantly high correlations (*R* > 0.70 and *p* < 0.05) were considered highly correlated.

In order to investigate the functional gene distribution of significant gene expression profiles and highly correlated modules, KEGG and GO enrichment analyses were jointly conducted. Significantly enriched KEGG pathways and GO terms were selected at a threshold of *p* < 0.05. A pathway-gene interaction network of significant pathways and important genes was constructed and visualized using Cytoscape v3.9.0 [83]. To observe relationships between genes and identify hub genes that have high connectivity with other genes, the genes of highly correlated modules mapped to the STRING v11.5 database [84] were selected to construct PPI networks. The PPI network was visualized by Cytoscape software.

### 4.4. Metabolomics Analysis

A total of 24 liver samples (six samples from each group) were used to extract metabolites (Figure 12C). Each liver sample was firstly ground in liquid nitrogen, and 100 mg of liver powder was placed in 500 μL of 80% aqueous methanol solution. After mixing well, the solution was placed on ice for 5 min and centrifuged at 15,000× *g* for 20 min at 4 °C. The supernatant was extracted by adding mass spectrometry-grade water until the aqueous methanol concentration reached 53%, then centrifuged at 15,000× *g* for 20 min at 4 °C. The second centrifugation of supernatant was transferred to autosampler vials for LC-MS analysis. LC-MS analysis was performed using a Vanquish UHPLC system (Thermo Fisher, Karlsruhe, Germany) coupled with an Orbitrap Q Exactive^TM^ HF-X mass spectrometer (Thermo Fisher, Germany). Details of the UHPLC system and mass spectrometer conditions are summarized in Table S4. The QC samples were composed of mixing equal aliquots of 24 liver samples, and the blank sample was 53% aqueous methanol solution. The QC and blank samples were also performed using the aforementioned protocol, then used for LC-MS analysis.

The acquired raw LC-MS data files were converted into mzXML format using Proteowizard v3.0 [85]. Peaks identification, filtration, and alignment were performed using XCMS program in R v4.0.2. In order to compare arrays, the peak areas of all data were batch normalized using the peak area of internal standard. After filtering the molecular formula error >30 ppm, the precise molecular weight was confirmed and applied in the metabolite identification. The classification and annotation of metabolites were identified by the Human Metabolome Database (HMDB) and KEGG database.

OPLS-DA is a useful algorithm that effectively reduces the model complexity and enhances the explanatory power of the model to evaluate the variation between groups. The reliability of the OPLS-DA model was assessed by permutation test. The DEMs were screened according to the variable importance in projection (VIP) scores obtained from the OPLS-DA analysis (VIP > 1 and *p* < 0.05). A KEGG enrichment analysis was conducted to investigate the metabolite function distribution and select significantly enriched KEGG pathways with a threshold of *p* < 0.05.

### 4.5. Integrated Analysis of Transcriptomics and Metabolomics

All DEGs and DEMs were used for integrated a KEGG pathway enrichment analysis. KEGG pathways with a threshold of *p* < 0.05 were considered as significantly enriched. The common significantly enriched pathways were constructed and mapped to DEMs that showed the relationship between metabolites and KEGG pathways using a Sankey plot.

### 4.6. qRT-PCR Validation for Key Genes

To validate the transcriptomics data, 10 hypoxia-associated genes were randomly selected for qRT-PCR. Specific primers (Table S5) of the target genes were designed using a Primer-BLAST webtool from NCBI (https://www.ncbi.nlm.nih.gov/tools/primer-blast/; accessed on 21 September 2022) and were synthesized by Genewiz Company (Guangzhou, China). The qRT-PCR mix contained 8.2 µL of nuclease-free ddH_2_O, 10 µL of 2 × PerfectStar^®^ Green qPCR SuperMix (TransGen Biotech, Beijing, China), 0.4 µL of each primer (10 µM) and 1 µL of cDNA. The qRT-PCR was performed in a Light Cycler 96 (Roche, Penzberg, Germany) using the following program: 30 s of denaturation at 94 °C, followed by 40 cycles of 94 °C for 5 s and 60 °C for 30 s. All qRT-PCRs were performed in triplicate. The housekeeping gene β-actin was used as an internal reference [86], and the relative expression of target genes was calculated using the comparative threshold (CT) method (2^−∆∆CT^) [87]. The qRT-PCR results were analyzed and visualized using GraphPad Prism v8.0.2 software (GraphPad Software, San Diego, CA, USA).

## 5. Conclusions

In this study, we mainly investigated transcriptomic and metabolic changes related to signal transduction, cell growth and death, carbohydrate metabolism, amino acid metabolism, and lipid metabolism in the liver of *T. ovatus* in response to acute hypoxic stress and re-oxygenation. During acute hypoxic stress, the cell cycle arrest, pro-apoptosis, and anti-apoptosis processes were enhanced. Cell cycle arrest could provide enough time to repair DNA damage, thereby maintaining genome stability and reducing energy consumption. Simultaneously strengthening pro-apoptosis and anti-apoptosis helped to reduce damaged signal-induced adverse impacts. After re-oxygenation 12 h, continuous apoptosis was induced that was in favor of cellular function and tissue repair. Furthermore, energy metabolism-related biological pathways, including glycolysis, gluconeogenesis, liver glycogen synthesis, aspartic acid metabolism, fat mobilization, and fatty acid biosynthesis, were enhanced to meet energy requirements and maintain intracellular homeostasis after acute hypoxic stress. After re-oxygenation, the energy metabolism strategy mainly shifted from anaerobic metabolism (glycolysis) during hypoxia to aerobic metabolism (fatty acid β-oxidation and TCA cycle), which may be an important adaptation mechanism for *T. ovatus*. These results, combined with the behavioral response of *T. ovatus* to hypoxic stress and previous studies, showed that hypoxia 6 h was a critical period of juveniles for metabolism alteration and cell homeostasis maintenance. Re-oxygenation intervention should be implemented within 6 h of hypoxic stress to avoid causing irreversible damage to *T. ovatus*, further avoiding mass mortality and serious economic losses. Furthermore, the re-oxygenation process should be continued for longer than 24 h to fully recover to normal. In conclusion, this study thoroughly examined the molecular mechanism of *T. ovatus* juveniles’ response to acute hypoxic stress at the transcriptomic and metabolic levels, which is conducive to the molecular breeding of hypoxia-tolerant cultivars and provides a scientific critical period for coping with acute hypoxic stress during the culture period. However, additional study with more experimental methodologies and techniques (single cell RNA-Seq, proteomics, gene knockdown, flow cytometric analysis, tissue sections, physiological and biochemical parameters, etc.) is clearly warranted to further explore heterogeneity in liver cell populations, key gene functions, severity of oxidative stress, molecular mechanism in response to hypoxia, and re-oxygenation duration for complete recovery of *T. ovatus*.

## Figures and Tables

**Table 1:** Transcriptome sequencing data and quality assessment of *Trachinotus ovatus*.

Sample Name	Hy0			Hy1			Hy6			Ro12		
	Hy0-1	Hy0-2	Hy0-3	Hy1-1	Hy1-2	Hy1-3	Hy6-1	Hy6-2	Hy6-3	Ro12-1	Ro12-2	Ro12-3
Raw reads (×10^6^)	39.06	43.39	38.85	40.63	41.55	40.95	42.33	41.99	41.23	41.33	59.29	44.58
Clean reads (×10^6^)	38.43	42.74	38.28	39.97	40.83	40.39	41.70	41.23	40.73	40.74	58.25	43.53
Clean reads rate (%)	98.39	98.50	98.53	98.38	98.27	98.63	98.51	98.19	98.79	98.57	98.25	97.64
Raw bases (×10^9^ bp)	5.86	6.51	5.83	6.10	6.23	6.14	6.35	6.30	6.18	6.20	8.89	6.69
Clean bases (×10^9^ bp)	5.73	6.38	5.72	5.97	6.09	6.03	6.23	6.15	6.08	6.09	8.70	6.49
GC content (%)	49.25	49.00	49.22	49.29	49.09	49.44	49.64	49.78	49.45	49.69	49.05	49.87
Q20 (%)	97.80	97.63	97.68	97.68	97.65	97.69	97.73	97.72	97.90	97.89	97.41	97.74
Q30 (%)	93.79	93.37	93.50	93.54	93.45	93.50	93.63	93.61	94.00	94.02	92.93	93.69
High-quality reads (×10^6^)	38.41	42.72	38.27	39.94	40.81	40.37	41.67	41.21	40.71	40.72	58.22	43.51
Total mapped (×10^6^)	35.83	40.02	35.80	37.46	38.15	38.06	39.18	36.97	38.35	37.54	54.07	40.79
Total mapped rate (%)	93.29	93.67	93.57	93.78	93.48	94.28	94.03	89.73	94.20	92.20	92.87	93.74
Unique mapped (×10^6^)	34.09	37.80	33.91	35.53	36.41	35.93	37.04	35.36	36.57	35.80	51.30	38.51
Unique mapped rate (%)	88.75	88.50	88.62	88.95	89.22	89.01	88.90	85.81	89.84	87.92	88.11	88.51

Note. Q20: correct recognition rate of the bases above 99%; Q30: correct recognition rate of the bases above 99.9%. High-quality reads: clean reads of removed rRNA mapped reads. Total mapped: high-quality reads totally mapped to reference genomic sequence. Unique mapped: high-quality reads uniquely mapped to reference genomic sequence.

**Table 2:** Key genes and metabolites related to signal transduction, cell growth and death, carbohydrate metabolism, amino acid metabolism, and lipid metabolism in response to acute hypoxic stress and re-oxygenation.

Genes/Metabolites	Description	Log2 (Fold Change)			
		Hy0 vs. Hy1	Hy0 vs. Hy6	Hy0 vs. Ro12	Hy6 vs. Ro12
Signal transduction					
*FOS*	proto-oncogene c-Fos	9.16 **	7.05 **	−0.18	−7.22 **
*JUN*	proto-oncogene c-Jun	6.43 **	5.03 **	0.08	−4.95 **
Cell cycle arrest					
*GADD45B*	growth arrest and DNA damage-inducible protein GADD45 beta	5.17 **	3.42 **	1.70 **	−1.72
*CDKN1A*	cyclin-dependent kinase inhibitor 1A	1.18 **	1.52 **	0.25	−1.28 *
Pro-apoptosis (intrinsic pathway)					
*BAX*	Bcl-2-associated X	0.62	1.25 **	0.21	−1.04 *
*CYC-B*	cytochrome c-b	2.13 **	4.73 **	1.39 *	−3.35 **
*CASP3*	caspase-3	−0.42	0.64 *	0.97 **	0.34
Pro-apoptosis (extrinsic pathway)					
*TNFRSF10A*	tumor necrosis factor receptor superfamily member 10A	1.84 **	1.72 **	0.15	−1.57 **
Pro-apoptosis (glutathione metabolism)					
*GPX7*	glutathione peroxidase 7	−1.37	−2.92 **	0.00	2.92 *
*GGT5*	glutathione hydrolase 5	0.59	1.34 *	1.91 **	0.57
Glutathione	–	−2.40 ^#^	−1.67 ^#^	0.58	2.25 ^#^
L-Glutamic acid	–	−0.63 ^#^	−0.90 ^#^	−0.63 ^#^	0.27
γ-Glutamylcysteine	–	3.16 ^#^	1.38	0.06	−1.32
L-Cysteine	–	0.31	1.17	2.93 ^#^	1.76 ^#^
Pro-apoptosis (sphingosine metabolism)					
*CERS6*	ceramide synthase 6	0.30	−1.14	1.51 *	2.65 **
Sphingosine	–	1.28	0.60	3.20 ^#^	2.60 ^#^
Anti-apoptosis					
*MCL1*	myeloid leukemia cell differentiation protein Mcl-1	1.48 **	1.77 **	0.07	−1.70 **
*HSP70*	heat shock protein 70	8.28 **	9.83 **	2.00	−7.83 **
*MDM2*	murine double minute 2	0.47 *	1.32 **	−0.41	−1.72 **
Glycolysis					
*HK1*	hexokinase 1	1.42 **	2.28 **	0.95	−1.33 *
*PFKL*	ATP-dependent 6-phosphofructokinase, liver type	−0.08	2.05 **	0.86 **	−1.19 *
*ALDOA*	fructose-bisphosphate aldolase A	1.52 **	3.22 **	1.40 **	−1.82 **
*ALDOCB*	fructose-bisphosphate aldolase C-B	0.95 **	2.86 **	1.07 **	−1.79 **
*GAPDH-2*	glyceraldehyde 3-phosphate dehydrogenase isoform 2	0.50	2.01 **	1.30 **	−0.71 *
*PGAM1*	phosphoglycerate mutase 1	1.27 *	3.16 **	2.13 *	−1.03
*ENO1*	alpha-enolase	−0.15	1.97 **	1.55 **	−0.42
*LDHA*	lactate dehydrogenase A	2.73 **	4.16 **	1.44 **	−2.71 **
Lactate transport					
*SLC16A3*	solute carrier family 16 member 3	3.25 **	4.44 **	1.56	−2.88 **
Gluconeogensis					
*G6PC*	glucose-6-phosphatase	2.02 **	1.29 **	−0.26	−1.56 **
*PCK1*	cytosolic phosphoenolpyruvate carboxykinase 1	4.95 **	4.70 **	1.39 *	−3.31 **
Liver glycogen synthesis					
*GYG2*	glycogenin-2	0.01	1.22 **	−0.49	−1.71 **
*GYS2*	glycogen synthase, liver	0.18	0.86 **	0.39	−0.47
*PHKA2*	phosphorylase b kinase regulatory subunit alpha, liver isoform	−0.61	−1.85 **	−0.95 *	0.90
*PYGL*	glycogen phosphorylase, liver form	−0.72	−1.42 *	−0.59	0.83
Amino acid metabolism					
*GOT2*	L-aspartic acid aminotransferase 2	0.74 *	1.62 **	0.29	−1.33 **
L-Aspartic acid	–	−0.37 ^#^	−0.75 ^#^	−0.56 ^#^	0.19
Fat mobilization					
*PNPLA2*	patatin-like phospholipase domain-containing protein 2	0.90 **	1.83 **	−0.56	−2.39 **
*LIPE*	hormone-sensitive lipase-like	0.98 *	1.90 **	−0.80	−2.69 **
Fatty acid biosynthesis					
*ACSL4*	long-chain-fatty-acid-CoA ligase 4	2.19 *	4.09 **	−4.62 **	−0.53
*ACLY*	ATP-citrate lyase	0.94	2.25 **	0.53	−1.72 *
Fatty acid β-oxidation					
*CPT1A*	carnitine O-palmitoyltransferase 1, liver isoform	0.35	−0.05	1.17 **	1.22 *
*CPT2*	carnitine O-palmitoyltransferase 2, mitochondrial	−0.80 *	−1.26 **	0.07	1.33 **
TCA cycle					
Oxoglutaric acid	–	0.48	0.30	1.01 ^#^	0.70

Note. Significance of DEGs (italic): * *p* < 0.05, ** *FDR* < 0.05. Significance of DEMs: ^#^ VIP > 1 and *p* < 0.05.

## Data Availability

The RNA-Seq raw data have been submitted to the Short Read Archive (SRA) data library under accession number PRJNA996362 through this address (https://www.ncbi.nlm.nih.gov/bioproject/PRJNA996362; accessed on 19 July 2023). The metabolomics data reported in this paper have been deposited in the OMIX, China National Center for Bioinformation/Beijing Institute of Genomics, Chinese Academy of Sciences (https://ngdc.cncb.ac.cn/omix/submitList: accession number: OMIX005161; accessed on 29 October 2023).

## Supplementary Materials

The supporting information can be downloaded at: https://www.mdpi.com/article/10.3390/ijms25021054/s1.
