# Supplementary material for: Integrated Transcriptomics and Metabolomics Reveal Changes in Cell Homeostasis and Energy Metabolism in Trachinotus ovatus in Response to Acute Hypoxic Stress
Source: Int J Mol Sci. 2024 Jan 15;25(2):1054. doi: 10.3390/ijms25021054 (PMC10815975; doi:10.3390/ijms25021054)
Supplement: Supplementary file 1 [file ijms-25-01054-s001.zip › Supplementary Figures S1-S5 and Tables S1-S5.pdf]

Supplementary Figures and Tables

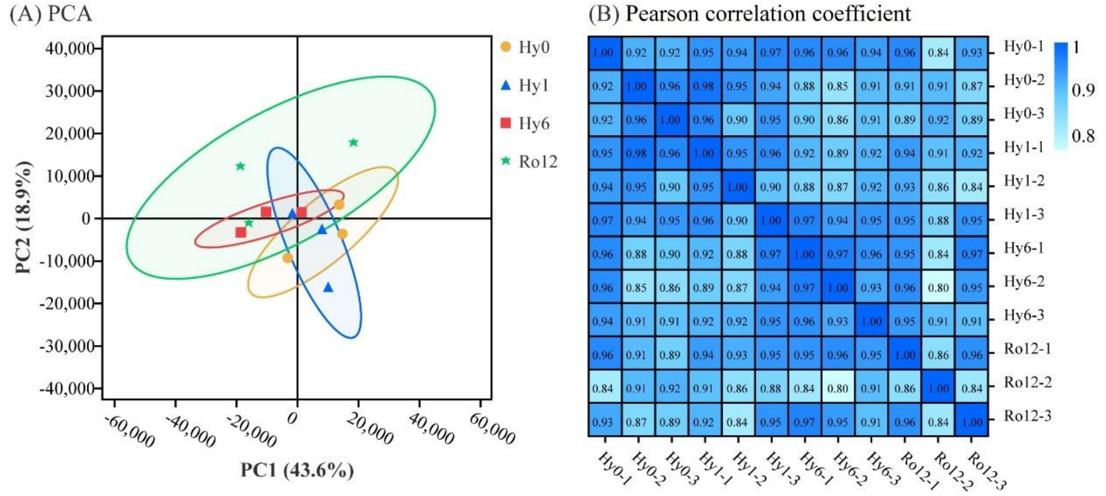

Figure S1. (A) Principal components analysis (PCA) and (B) Pearson's correlation coefficient between samples.

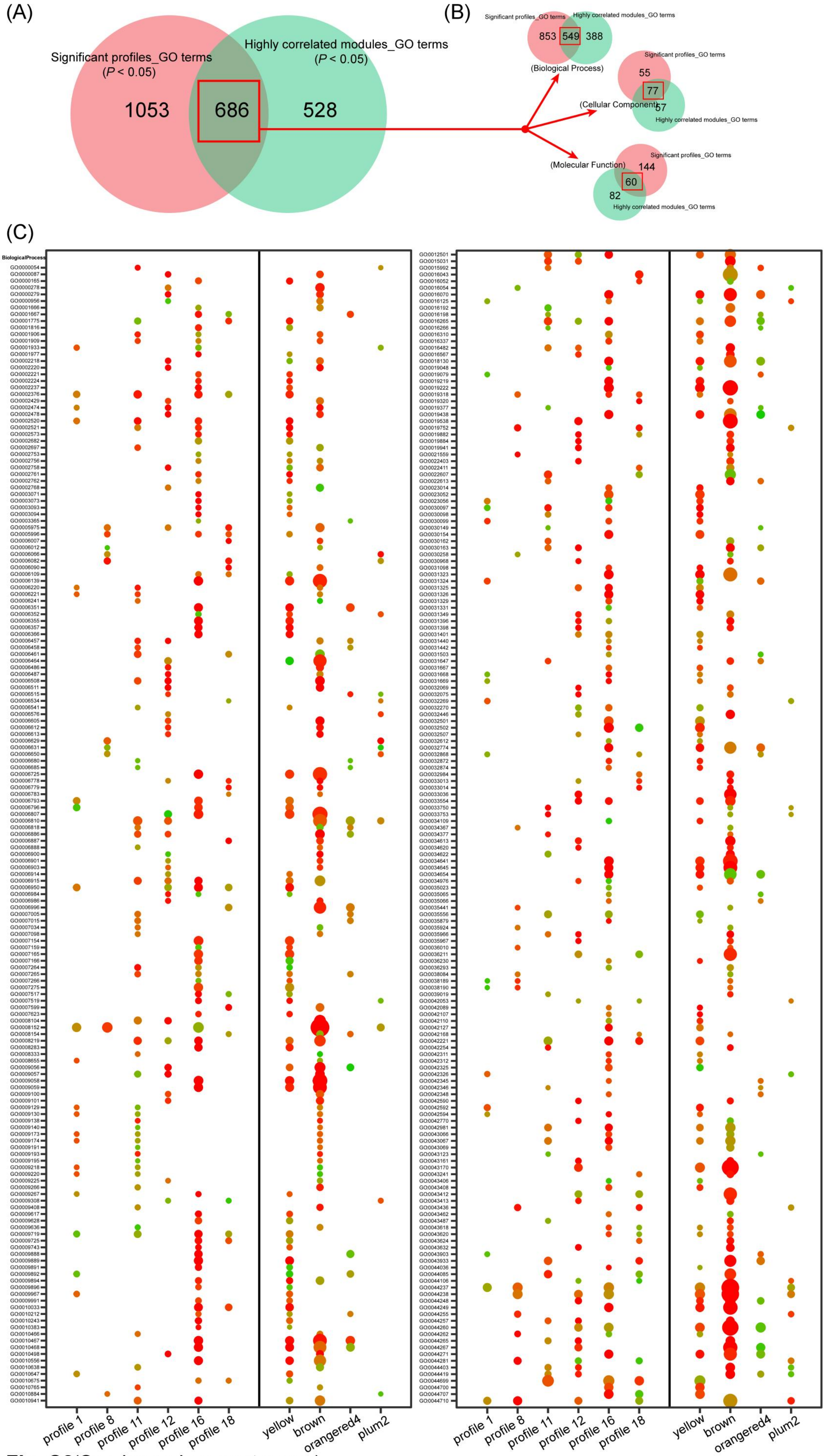

Fig. S2(See lenged on next page.)

(See **Fig. S2** on previous page.)

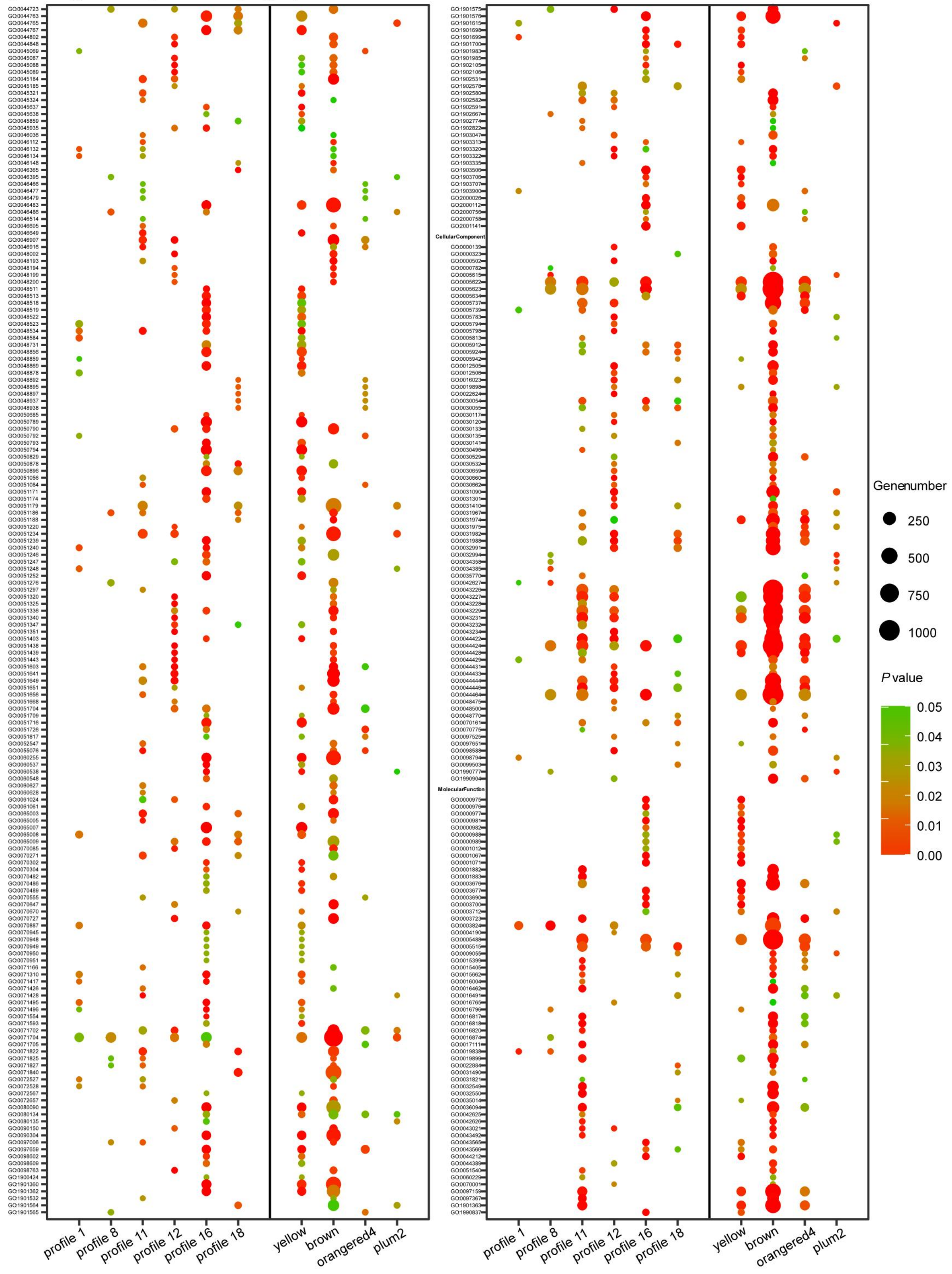

Figure S2. GO enrichment analysis. (A) Venn diagram of significant GO terms (P < 0.05) between six significant gene expression profiles (red circles) and four highly correlated modules (green circles). (B) Venn diagrams of significant GO term classifications. (C) The 686 common GO terms of significant profiles and highly correlated modules.

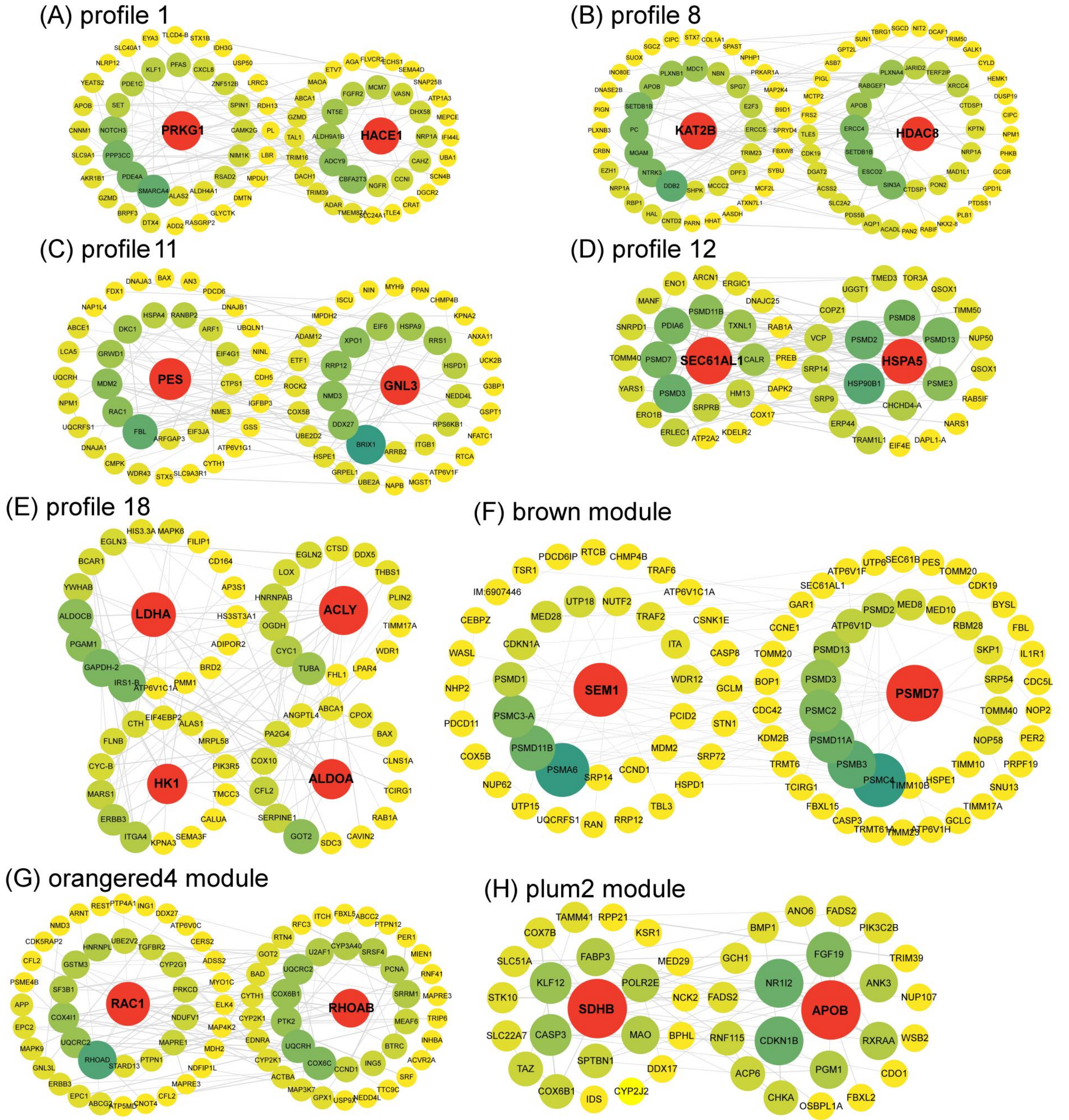

Figure S3. Protein-protein interaction (PPI) network analysis. The visualization of the first 100 interactions of profile 1 (A), profile 8 (B), profile 11 (C), profile 12 (D), profile 18 (E), brown module (F), orangered4 module (G), and plum2 module (H). The nodes represent genes; nodes size and color are related to the degree attribute of the PPI network; and larger size and deeper color indicate higher connectivity between genes. The edges indicate the connectivity between genes, the size of the edges is correlated with the combined score attribute, with a larger size representing a higher combined score.

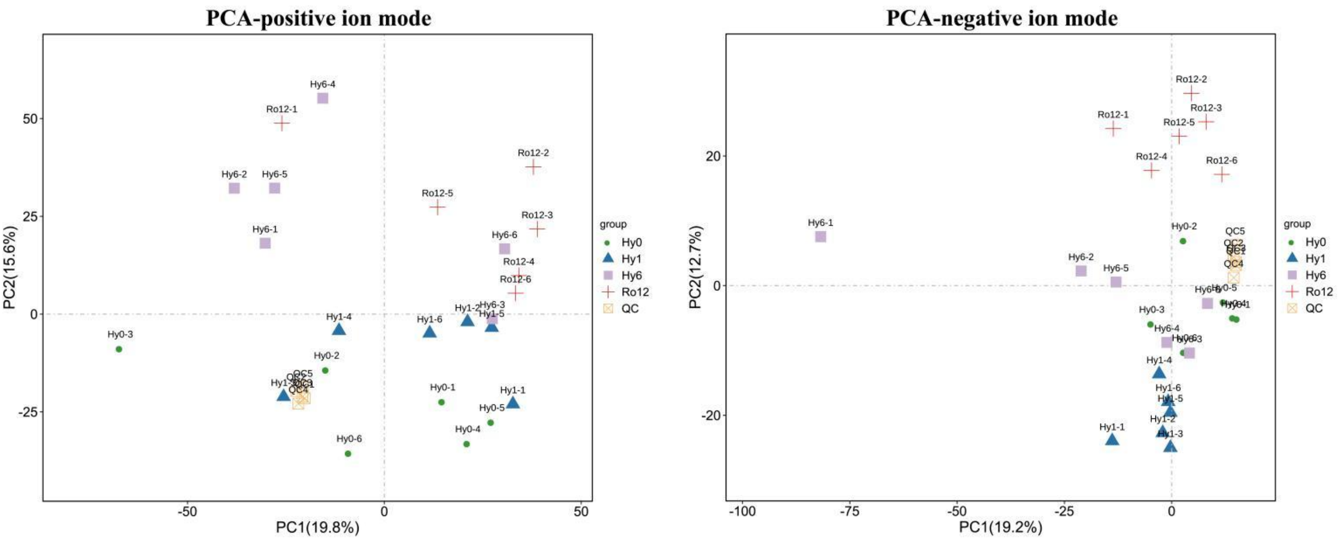

Figure S4. PCA analysis between treatment groups and QC sample under positive and negative ion modes.

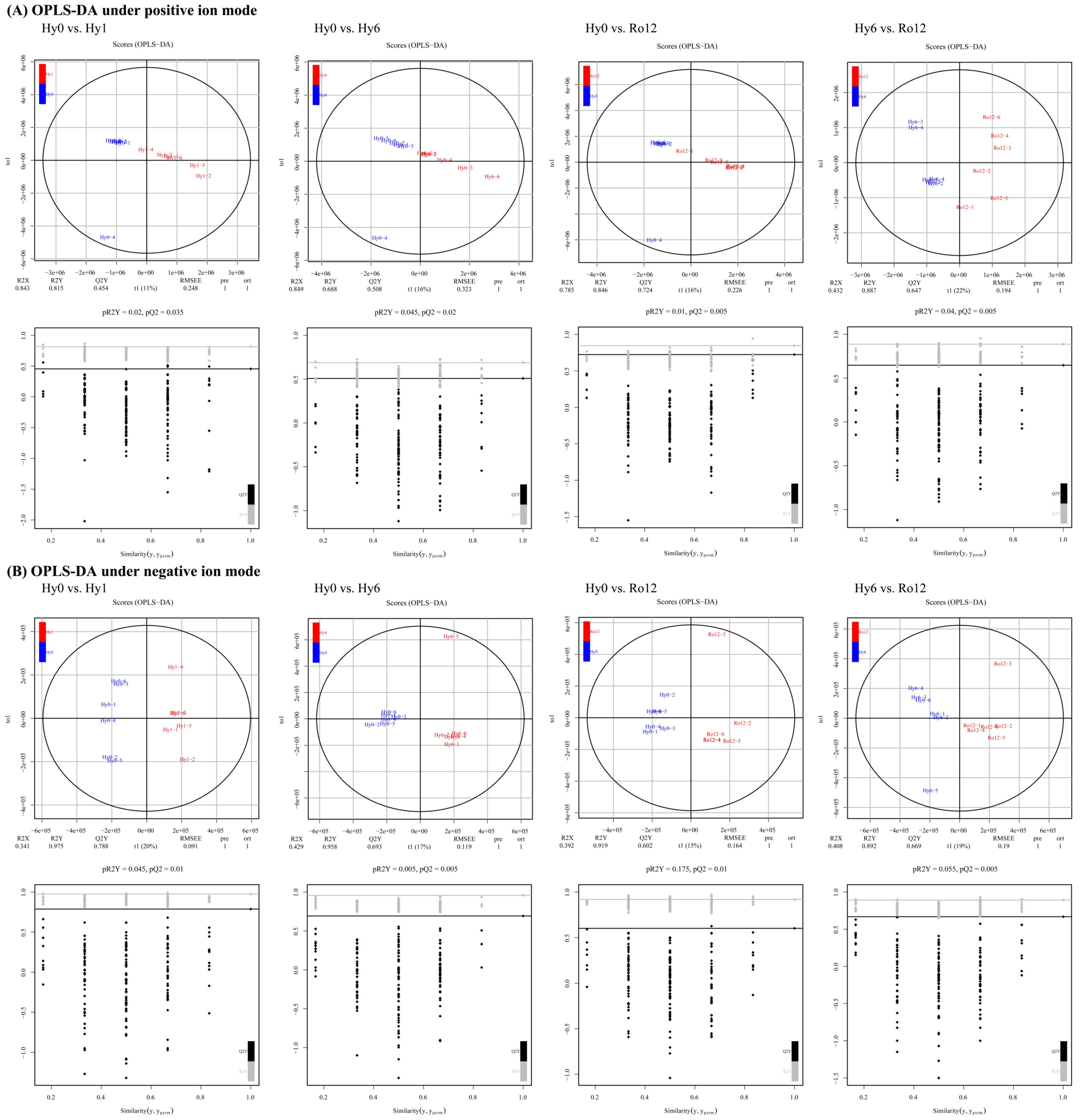

Figure S5. Orthogonal projection to latent structure-discriminant analysis (OPLS-DA) and permutation test under positive (A) and negative (B) ion modes.

Table S1 Specific gene number of 18 weighted gene co-expression network analysis (WGCNA) modules.

Module	Gene number	Module	Gene number	Module	Gene number
**brown**	**2,215**	darkturquoise	432	darkorange	153
lightsteelblue1	1,999	darkorange2	418	violet	137
brown4	1,315	darkmagenta	366	bisque4	93
black	615	darkred	356	**plum2**	**89**
lightpink4	502	**yellow**	**344**	thistle1	81
**orangered4**	**433**	green	335	grey	5

Table S2 Identification of metabolites under positive and negative ion modes.

Mode	Total	MS1	MS2	Known (MS1+ MS2)	Unknown
Positive ion mode	4,114	721	368	1,089	3,025
Negative ion mode	1,811	419	104	523	1,288

Table S3 The parameters of orthogonal partial least squares-discriminant analysis (OPLS-DA) model in Hy0 vs. Hy1, Hy0 vs. Hy6, Hy0 vs. Ro12, and Hy6 vs. Ro12 groups.

Mode	Group	R^2X	R^2Y	Q^2Y
Positive ion mode	Hy0 vs. Hy1	0.843	0.815	0.454
	Hy0 vs. Hy6	0.849	0.688	0.508
	Hy0 vs. Ro12	0.785	0.846	0.724
	Hy6 vs. Ro12	0.432	0.887	0.647
Negative ion mode	Hy0 vs. Hy1	0.341	0.975	0.788
	Hy0 vs. Hy6	0.429	0.958	0.693
	Hy0 vs. Ro12	0.392	0.919	0.602
	Hy6 vs. Ro12	0.408	0.892	0.669

Table S4 Specific primer sequences of ten randomly selected genes and one reference gene for qRT-PCR validation.

Condition	Parameters
**UHPLC systems**	
Column temperature	40℃
Chromatographic separation	Hypesil Gold column (C18)
Flow rate	17-min linear gradient with 0.2 $mL{\cdot}min^{-1}$
Positive ion mode: mobile phase A	0.1% formic acid in water
Positive ion mode: mobile phase B	Methanol
Negative ion mode: mobile phase A	5 mM ammonium bicarbonate in water (pH = 9.0)
Negative ion mode: mobile phase B	Methanol
**Mass spectrometer**	
Type	Electrospray ion source (ESI)
Electrospray voltage of positive ion mode (ESI+)	3.2 kV
Electrospray voltage of negative ion mode (ESI-)	2.5 kV
Ion source temperature	320℃
Sheath gas flow rate	40 arb
Aux gas flow rate	10 arb
Full scan	70,000 resolution
Scan range	80–1,000 m/z
Collision voltage	30 eV

Table S5 Specific primer sequences of ten randomly selected genes and one reference gene for qRT-PCR validation.

Gene_id	Gene_symbol	Forward primer (5′–3′)	Reverse primer (5′–3′)
/	β-actin*	CGTGCGTGACATCAAGGAGAA	AAGGAAGGAAGGCTGGAAGAGG
SCSFRI_TO_T_00039016.path1	*FOS*	GTCACCAGCGGGATCTTACTC	GAAATGGCTGTGACAGTGGG
SCSFRI_TO_T_00009724.path1	*BAX*	AGGGTGGTCGCACTGTTCTA	CAAGAAGACCCCCAACGTCT
SCSFRI_TO_T_00117137.path1	*MCL1*	ACCGATGTGTCGAGTTGTCC	TTTTCCAAAACGCCGTCCAC
SCSFRI_TO_T_00065768.path1	*CYC-B*	GGTCTGTTTGGACGCAAGAC	CTTCTTTTTGATGCCGGCGAA
SCSFRI_TO_T_00046973.path1	*HSP70*	ATACCTCGGGCAACGTTCAG	GTCCACCGCAGATACGTTCA
SCSFRI_TO_T_00135876.path1	*ALDOA*	AGAAGACGGATGACGGCAAG	GTCCAGACCTTGAGTGGTCG
SCSFRI_TO_T_00089332.path1	*LDHA*	CGTCAAGTACAGCCCCAACT	TCTCCAATGATCCAGCCGTG
SCSFRI_TO_T_00015468.path1	*PCK1*	AACAACTGGCCCTGTAACCC	TTGGCGATACGTGAGGCAAT
SCSFRI_TO_T_00000587.path1	*PNPLA2*	GCCAAGGTGTCTGAGATGCT	GTGTCGGTACACCTCACCAG
SCSFRI_TO_T_00095046.path1	*ACSL4*	GCGAGTTTGGCAGTGGATTG	AGCAATAGCCTCCTCTCCGA

*Reference: Chen et al., 2017.
